# A Scoping Review of Neurotoxic and Behavioral Outcomes Following Polychlorinated Biphenyl (PCB) Exposure in Post-Weaned Rodents

**DOI:** 10.3390/ijms262210829

**Published:** 2025-11-07

**Authors:** Nicole M. Breese, Sophia G. Heim, Riley J. Samuelson, Hans-Joachim Lehmler

**Affiliations:** 1 Department of Occupational and Environmental Health, College of Public Health, The University of Iowa, Iowa City, IA 52242, USA; nicole-breese@uiowa.edu (N.M.B.); sophia-heim@uiowa.edu (S.G.H.); 2 Interdisciplinary Graduate Program in Human Toxicology, The University of Iowa, Iowa City, IA 52242, USA; 3 Hardin Library for the Health Sciences, University of Iowa Libraries, The University of Iowa, Iowa City, IA 52242, USA; riley-samuelson@uiowa.edu

**Keywords:** behavioral outcomes, cognitive function, dopaminergic system, environmental neurotoxicants, large language models, neurochemical alterations, neurotoxicity, oxidative stress, polychlorinated biphenyls, post-weaning exposure, rodent models

## Abstract

Polychlorinated biphenyls (PCBs) are persistent organic pollutants associated with neurodevelopmental toxicity, yet the effects of exposure during adolescence and adulthood remain underexplored. This scoping review evaluates the neurotoxic outcomes of post-weaning PCB exposure in rodent models. A comprehensive literature search was conducted across PubMed, Embase, and Scopus. Studies were screened according to PRISMA guidelines. Articles were included if they reported neurotoxic or behavioral outcomes in mice or rats exposed to PCBs during post-weaning stages. Thirty-five studies met the inclusion criteria, encompassing a variety of PCB congeners and mixtures administered via oral, inhalation, or intraperitoneal routes. Reported neurotoxic outcomes included histological and morphological brain changes, oxidative stress, disrupted calcium signaling, altered neurotransmitter systems, apoptosis, and gene expression alterations. These outcomes were assessed using diverse methodological approaches, including immunohistochemistry, biochemical assays, and gene expression profiling. Behavioral outcomes affected by PCB exposure included locomotion, anxiety-like behavior, learning and memory, motor coordination, and cognitive flexibility. Effects were often exposure-specific and sex-dependent, with limited female-focused studies and integrative molecular-behavioral assessments. These findings highlight the broad neurotoxic potential of PCBs following adolescent or adult exposure and underscore the need for further mechanistic, sex-specific research to inform health risk assessment and regulatory policy.

## 1. Introduction

Polychlorinated biphenyls (PCBs) are a class of 209 synthetic chlorinated hydrocarbons that were manufactured for industrial and commercial products due to their electrical insulation properties and chemical stability [1]. Commercial PCB mixtures, sold in the United States (U.S.) under the trade name Aroclor, served as dielectric fluids in transformers and capacitors [1]. PCBs were also used as plasticizers and in lubricants, adhesives, and sealants in various building materials, such as caulking, floor finishes, and paints [1,2,3]. Although their manufacture was banned in the U.S. in 1979 under the Toxic Substances Control Act, PCBs continue to be pervasive in indoor environments [1]. PCB-containing caulks and sealants applied between the 1950s and 1970s continue to volatilize, emitting vapors that accumulate in indoor air and dust [4,5]. Studies in schools in the U.S. and Europe have documented the presence of PCBs in indoor air, raising significant concerns for public health [6,7,8,9,10,11]. Moreover, PCBs persist in the environment due to their resistance to degradation [12] and continue to be released into the environment from legacy sources [13,14]. Importantly, PCBs are inadvertently generated as byproducts of manufacturing processes [13,14,15].

Due to their lipophilicity and environmental persistence, PCBs bioaccumulate and biomagnify in the food chain, particularly in the fatty tissues of fish and other animals [16,17]. The bioaccumulation of PCBs is congener-dependent because physicochemical properties, three-dimensional structure, uptake, biotransformation, and excretion vary by congener [18,19,20]. Human exposure to PCBs occurs through ingestion of contaminated food and dermal contact [2,21]. Evidence from laboratory [22,23,24,25,26,27,28,29,30,31,32] and epidemiological studies [33,34,35,36,37] demonstrates that inhalation is an important route of current human PCB exposure. Human biomonitoring studies have detected PCBs in serum, adipose, and brain tissues across populations regardless of age, race, and socioeconomic status [38,39]. PCBs are a significant public health concern with well-documented toxic effects, including, but not limited to, carcinogenicity [40,41], immunotoxicity [42,43], reproductive toxicity [44,45], developmental toxicity [46,47], neurotoxicity [48,49], endocrine disruption [50,51], and cardiovascular impacts [52,53]. Epidemiological studies have established associations between PCB exposure and a range of adverse neurological outcomes, including decreased cognitive function and mood dysregulation, such as anxiety and depression [39,54].

Individual PCB congeners can be categorized into two functional classes based on structural and toxicological properties. Dioxin-like PCBs, which include congeners such as PCB 77 or PCB 126, can act like dioxins in the body [55]. Like dioxins, dioxin-like PCBs exert their toxicity through activation of the aryl hydrocarbon receptor (AhR) [56], with developmental effects as the most sensitive toxic endpoint [57]. In contrast, non-dioxin-like PCBs, such as PCB 180, act via AhR-independent pathways, including disruption of calcium homeostasis [58]. Non-dioxin-like PCBs have different toxicological activity, with more sensitivity in the liver and thyroid [59]. AhR-independent mechanisms involve activation of several nuclear receptors, such as the constitutive androstane receptor (CAR) and pregnane X receptor (PXR), both of which regulate the expression of genes involved in xenobiotic metabolism [60]. These mechanistic distinctions are essential for understanding the congener-specific risks associated with environmental PCB exposure. Other receptors also contribute to the diverse toxicological profiles of individual congeners. Ryanodine receptors (RyRs) are critical targets of non-dioxin-like PCBs, particularly ortho-substituted congeners, leading to dysregulated calcium signaling and altered neuronal excitability [61,62]. Estrogen receptors (ERs) and androgen receptors (ARs) may be modulated by specific hydroxylated PCB metabolites, acting as endocrine disruptors that interfere with hormone signaling [63,64]. Additionally, peroxisome proliferator-activated receptors (PPARs) are implicated in PCB-induced metabolic dysfunction, particularly through activation by PCBs [65,66].

PCB metabolism is a complex process involving multiple pathways [67,68,69]. PCBs undergo biotransformation through Phase I and Phase II metabolic pathways, primarily mediated by hepatic enzymes [70]. In Phase I metabolism, cytochrome P450 monooxygenases (CYPs), such as CYP1A and CYP2B isoforms, catalyze the oxidation of PCBs to form hydroxylated metabolites (OH-PCBs) [71,72,73,74]. PCB metabolism is congener-specific and strongly influenced by the number and position of chlorine atoms [75]. Non-ortho and mono-ortho-substituted PCBs are more readily metabolized than fully ortho-substituted or higher chlorinated congeners [76,77]. Some OH-PCBs can undergo further oxidation to form dihydroxylated PCBs [74], which may oxidize to form reactive PCB quinones, capable of redox cycling and inducing oxidative stress [78,79]. Phase II metabolism involves the conjugation of hydroxylated PCBs with polar functional groups to increase water solubility and promote excretion [80,81]. This includes glucuronidation by UDP-glucuronosyltransferases (UGTs) [82], sulfation by sulfotransferases (SULTs) [83], and glutathione conjugation via glutathione-S-transferases (GSTs) [84]. Additional processes include methoxylation of hydroxylated metabolites by O-methyltransferase (OMT) [85], and the formation of methyl sulfones via the mercapturic acid pathway [86]. Understanding these biotransformation pathways is crucial for interpreting differences in susceptibility and assessing the risk of neurotoxicity from PCB exposures.

While the neurodevelopmental toxicity of PCBs has been extensively studied in the context of prenatal and lactational exposure [48,87,88,89], much less is known about the consequences following post-weaning PCB exposure. Additionally, most preclinical models focus exclusively on either behavioral or molecular outcomes, limiting the ability to draw mechanistic connections between PCB-induced changes in neurochemistry and observed functional impairments. Furthermore, the sex-specific effects of PCB exposure during critical windows of exposure remain underexplored, despite increasing evidence that males and females may differ in their vulnerability and response to neurotoxicants [90]. To summarize the available evidence, a scoping review of the literature was conducted, with a specific focus on PCB exposure during the post-weaning period and its associated neurotoxic effects.

## 2. Methods

### 2.1. Selection of Articles

#### 2.1.1. Search Strategy

**Figure 1:**
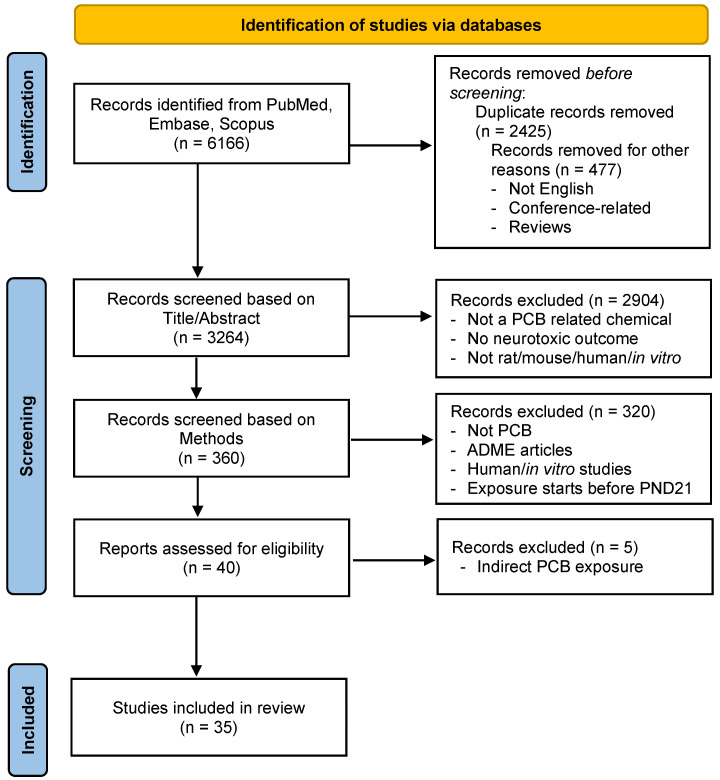
A Preferred Reporting Items for Systematic Reviews and Meta-Analysis (PRISMA) flow diagram for study identification, inclusion, and exclusion [88].

The search strategy was adapted from the Preferred Reporting Items for Systematic Reviews and Meta-Analysis (PRISMA) best-practice guidelines and developed with a librarian trained in scoping reviews [91]. The scoping review, which complies with the PRISMA guidelines, was not preregistered. An outline of the search strategy, the inclusion and exclusion criteria, and the data extraction process is summarized in the flow chart shown in Figure 1. Briefly, a comprehensive search was conducted in PubMed, Scopus, and Embase using Boolean search terms that included combinations of “PCBs,” “neurotoxicity,” “mice,” “rats,” “adolescents,” and “adults” (see Supporting Information Table S1).

While the search strategy also included title/abstract search terms, a truncated form of the PubMed search with only controlled vocabulary (Medical Subject Headings) is as follows: (“Rats”[Mesh] OR “Murinae”[Mesh] OR “Mice”[Mesh]) AND (“Adolescent”[Mesh] OR “Child”[Mesh] OR “Minors”[Mesh] OR “Adult”[Mesh] OR “Middle Aged”[Mesh] OR “Aging”[Mesh] OR “Aged”[Mesh] OR “Aged, 80 and over”[Mesh] OR “Young Adult”[Mesh]) AND (“Polychlorinated Biphenyls”[Mesh] OR “Biphenyl Compounds”[Mesh] OR “Aroclors”[Mesh] OR “Dioxins and Dioxin-like Compounds”[Mesh] OR “Dioxins”[Mesh]) AND (“Attention Deficit Disorder with Hyperactivity”[Mesh] OR “Neurodevelopmental Disorders”[Mesh] OR “toxicity”[Subheading] OR “Neurocognitive Disorders”[Mesh] OR “Growth and Development”[Mesh] OR “Brain”[Mesh] OR “Central Nervous System”[Mesh] OR “Nervous System”[Mesh]). The searches were conducted in November 2023. These citations were imported into EndNote (Version 20.6, RRID:SCR_014001). The Bramer method [92] was employed to deduplicate references. Additional articles were excluded based on the predefined exclusion criteria, including non-English language, non-peer-reviewed work (e.g., editorials, commentaries), and irrelevant study types (e.g., reviews).

#### 2.1.2. Title and Abstract Screening

The title and abstract screening were performed in parallel using both manual screening by researchers and automated screening with ChatGPT 4o (OpenAI, San Francisco, CA, USA) [93]. Two researchers independently screened the titles and abstracts to identify studies involving mouse, rat, human, or in vitro subjects exposed to PCBs or related chemicals with a reported neurotoxic outcome. Disagreements were resolved through discussion or by a third reviewer when consensus could not be reached.

#### 2.1.3. Automated Screening Using ChatGPT

Given the complexity of this topic, which includes the large number of PCB congeners and mixtures, varying windows and routes of exposure, and diverse outcome measures, scoping reviews of this nature are inherently labor-intensive and time-consuming. To improve the rigor and scalability of this process, ChatGPT was utilized following a recently published method [93] to aid in literature screening and data extraction. Default temperature settings and standard conversation context were applied via the OpenAI web interface on a desktop computer (Intel Core i7 processor, 8 GB RAM, Windows 10, Google Chrome browser), with the “improve the model for everyone” feature disabled. No custom system prompts, external fine-tuning, or temperature adjustments were used. Each session was initialized with the same prompts to guide the model’s reasoning. ChatGPT was trained using a series of specific prompts (Table 1) to improve its ability to classify relevant studies [93]. First, ChatGPT was asked to define each category to ensure consistent classification. Subsequently, ChatGPT was asked to categorize each article into one or more of the following categories: (1) mouse, rat, human, or in vitro subjects, (2) PCB or related chemical exposure, (3) neurotoxic outcome, or (4) not related. ChatGPT was then prompted to classify each title and abstract, returning its classifications in a table format. ChatGPT was asked to use an “X” to indicate which categories applied to each article, with a brief explanation for each selection (Table 2). These results were transferred into Microsoft Excel for Microsoft 365 MSO (Version 2506, 64-bit, Windows) for further analysis.

#### 2.1.4. Analysis of Screened Articles

**Figure 2:**
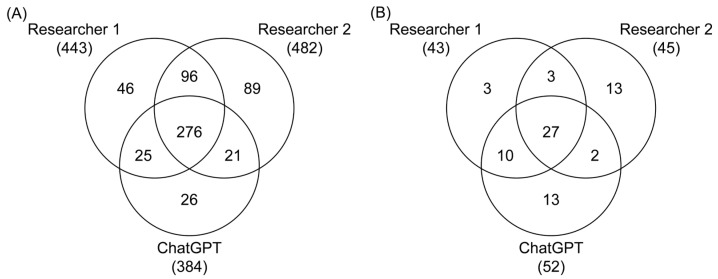
Venn diagram comparing article selections based on (**A**) title/abstract between the two researchers and ChatGPT and (**B**) methods section between the two researchers and ChatGPT. The total number of articles selected by each reviewer is in parentheses.

An article was selected for the next screening phase based on the classification of both researchers and ChatGPT. If only two of the three screeners (both researchers or one researcher and ChatGPT) selected an article, the article was re-evaluated by both researchers, with disagreements resolved by a third reviewer acting as a tiebreaker. Articles selected by only one screener were excluded from further screening (Figure 2).

### 2.2. Method Screening

#### 2.2.1. Manual Screening of Methods Sections

Two independent researchers manually screened the methods sections of the selected articles. The aim was to identify studies involving mice or rats exposed to PCBs starting on postnatal day 21 or later, with reported neurotoxic or behavioral outcomes. Discrepancies between the two researchers were resolved through discussion or by a third reviewer, who served as a tiebreaker if necessary.

#### 2.2.2. Automated Methods Screening Using ChatGPT

**Figure 3:**
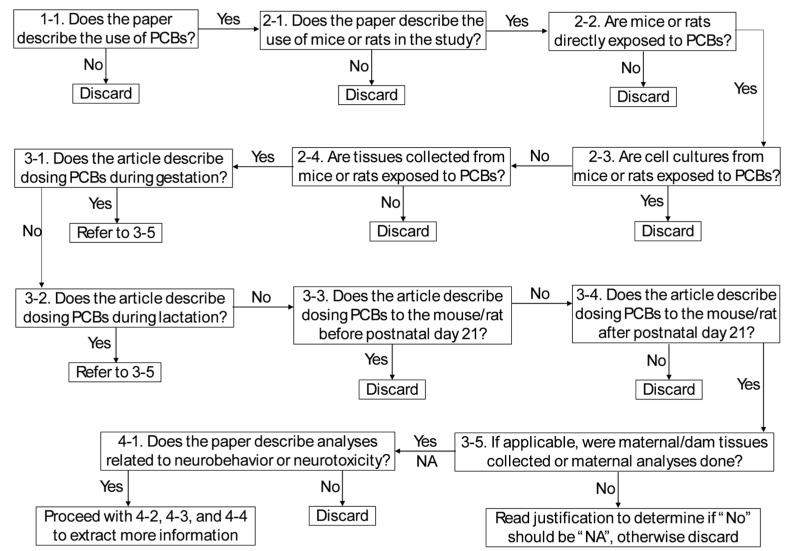
Decision tree guiding human reviewers in filtering ChatGPT-generated responses to the 14 yes/no questions used for the analysis of the methods sections.

Subsequently, ChatGPT was utilized to screen the methods sections. Four key criteria were evaluated: (1) PCB exposure, (2) study subject (mice or rats), (3) dosing timeframe (postnatal day 21 or later), and (4) neurotoxic outcomes. Fourteen yes/no questions were created (Table 3) to guide ChatGPT’s evaluation of each article’s methods. ChatGPT was prompted to answer each question in a table format, with explanations for each “Yes” or “No” answer (Table 4). These results were copied into an Excel sheet and filtered to identify studies that met the predefined criteria. To ensure consistency and reproducibility, a decision tree (Figure 3) was developed to guide reviewers in interpreting and verifying ChatGPT’s responses. This decision tree outlined the logical sequence by which reviewers determined whether a study should be included, excluded, or flagged for further discussion.

#### 2.2.3. Analysis of Methods Screening Results

An article was advanced to the full-text assessment if both researchers and ChatGPT identified the article as meeting the relevant criteria. If only two out of three screeners (both researchers or one researcher and ChatGPT) selected an article, the article was re-evaluated by both researchers. A third-party reviewer resolved any disagreements that arose as necessary. Articles selected by only one screener were excluded from further screening.

### 2.3. Full-Text Assessment

Prior to full-text assessment, a data extraction table was established to identify key information such as animal strain, sex, age, exposure compound(s), dose, route of administration, and behavioral or neurotoxic outcomes [94]. During full-text assessment, both researchers reviewed the entire article to extract key information. Five articles were identified as having indirect PCB exposure (e.g., through contaminated fish) and were excluded. Indirect exposure was defined as exposure resulting from naturally contaminated materials in which the PCB dose and congener profile could not be accurately determined or experimentally controlled. Such studies were excluded because the actual exposure conditions cannot be reproduced by other laboratories. The present review, therefore, focused on controlled experimental exposures in which PCB congeners or mixtures were deliberately administered via defined routes (e.g., oral gavage, inhalation, or intraperitoneal injection). From each included study, we extracted detailed information on study characteristics (author names, year of publication, country, and funding source), animal model features (species, strain, sex, age at exposure, and developmental stage), and exposure parameters (route, dose, duration, frequency, exposure window, and the specific PCB congeners or mixtures tested). Information on control conditions (e.g., vehicle-treated, untreated, or sham controls) was also recorded. Outcomes were classified as either neurotoxic (e.g., histological changes, alterations in neurotransmitter levels, or biomarkers of neurotoxicity) or behavioral (e.g., performance in learning and memory tasks, motor activity assessments, and anxiety-like behavior). Additional study design features were extracted, including sample size and methods of outcome assessment. Behavioral outcomes were grouped into broad domains, such as cognitive, motor, or anxiety-related, to enable synthesis across methodologies. All extracted information was organized into summary tables (Table A1, Table A2, Table A3, Table A4, Table A5, Table A6 and Table A7).

### 2.4. Limitations

Several limitations of the review process should be acknowledged. First, restricting the search to English-language publications may have led to the omission of relevant studies, particularly those published in non-indexed sources. The exclusion of gray literature and non-peer-reviewed studies may have further narrowed the scope. During title, abstract, and full-text screening, subjective decisions made by human reviewers and ChatGPT may have introduced bias, despite efforts to standardize the classification process. The application of strict exclusion criteria may have excluded studies with mixed exposures or partially relevant designs. Data extraction was complicated by inconsistent reporting of exposure doses, animal characteristics (e.g., strain, sex), and neurobehavioral outcomes, requiring simplification that may have obscured important nuances. As a scoping review, no formal assessment of study quality was conducted, and no quantitative synthesis was performed; thus, findings are descriptive and do not reflect effect sizes or dose–response relationships. Finally, focusing solely on post-weaning rodent models may limit generalizability to other developmental stages or species, and grouping diverse exposure paradigms may have masked important differences in congener-specific or temporal effects.

### 2.5. Statistical Analysis

The agreement of the human reviewers and ChatGPT was evaluated using Cohen’s and Fleiss’ kappa at the title/abstract and methods screening steps of the review process. Cohen’s kappa evaluates the agreement between two reviewers and was developed to consider the likelihood that raters may make guesses on some variables due to uncertainty [95]. Kappa values were calculated for each of the comparisons (Reviewer 1 & Reviewer 2, Reviewer 1 & ChatGPT, and Reviewer 2 & ChatGPT) to determine agreement between raters. Values > 0.21 are considered “Fair”, values > 0.41 are considered “Moderate”, values > 0.61 are considered “Substantial”, and values > 0.81 are considered “Almost Perfect”. Fleiss’ kappa is similar to Cohen’s and allows for more than two raters to be evaluated [96].

## 3. Results and Discussion

### 3.1. Article Selection

An outline of the search strategy, the inclusion and exclusion criteria established a priori, and the data extraction process are summarized in the flow chart shown in Figure 1. This search identified a total of 6166 citations. The Bramer method [92] was employed to remove 2425 duplicate articles. Subsequently, 477 additional articles were excluded based on the predefined exclusion criteria. This process resulted in 3264 articles being eligible for title and abstract screening, which was performed independently by two Reviewers and ChatGPT. During title and abstract screening, Researcher 1 identified 443 articles, Researcher 2 identified 482 articles, and ChatGPT identified 348 articles that met the inclusion criteria. This resulted in 276 articles selected by all three screeners, 142 articles selected by two reviewers or one reviewer and ChatGPT, and 161 selected by only one reviewer. These 161 articles were excluded from the review (Figure 2A). The 142 articles selected by two reviewers or ChatGPT were reevaluated by the two human reviewers, with a third reviewer serving as a tiebreaker. Of these articles, 84 were selected for inclusion, resulting in 360 articles proceeding to the methods screening phase. During method screening, Researcher 1 identified 43 relevant articles, Researcher 2 identified 45 articles, and ChatGPT identified 52 articles. This resulted in 27 articles being selected by all three screeners, and 44 articles underwent re-evaluation (Figure 2B), ultimately leading to 40 articles being chosen for full-text assessment. During full-text assessment, 5 articles were identified as having indirect PCB exposure (e.g., through PCB-contaminated fish [97,98,99,100] or food grown on PCB-contaminated sediment [101]) and were excluded, resulting in 35 articles selected for inclusion. Overall, the studies investigated outcomes primarily in male rats and mice with 24 studies examining only males, 5 investigating males and females, and 6 studies examining only female rodents. In addition to these 35 studies, other relevant literature is cited throughout the manuscript to provide background, methodological comparisons, or mechanistic insights.

Screening time was measured using a start–stop method, where each reviewer recorded the duration required to complete screening for each batch of abstracts or methods, and an average time was calculated for all articles. Researchers spent an average of 54.4 h screening titles and abstracts, while using ChatGPT reduced this time to about 12 h. During the methods screening stage, each researcher invested around 36 h, whereas ChatGPT required only about 9 h. These findings suggest that ChatGPT can significantly reduce time spent during the initial screening phases.

### 3.2. Inter-Reviewer Reliability Assessment

During the title and abstract screening stage, Reviewer 1 and Reviewer 2 demonstrated the highest percentage of agreement and Cohen’s kappa, while their agreement with ChatGPT was lower (Table 5). The fixed-marginal Fleiss’ kappa value for Reviewer 1, Reviewer 2, and ChatGPT was 0.73, with a 95% confidence interval of 0.68–0.77, indicating significant agreement and an overall agreement of 93.81%. During the methods screening stage, Reviewer 1 and ChatGPT showed the highest percent agreement and Cohen’s kappa, while agreement with Reviewer 2 was lower for both Reviewer 1 and ChatGPT (Table 6). Fleiss’ kappa for the method selection step was determined for Reviewer 1, Reviewer 2, and ChatGPT. There was an overall agreement of 91.85%, with a fixed-marginal kappa of 0.64 and a 95% confidence interval of 0.49–0.78, indicating moderate to significant agreement. Consistent with earlier research [93], our results support the integration of ChatGPT and similar large language models as effective tools for scoping review.

### 3.3. Neurotoxic Outcomes

This section provides a scoping review of the neurotoxic effects of PCBs observed in rodent models, focusing on histological, molecular, and biochemical endpoints, in the studies identified in the scoping review. Of the 27 studies reporting neurotoxic outcomes, 22 studies examined only males, 2 investigated males and females, and 3 studies examined only female rodents. Studies examined a range of exposure routes (oral, inhalation, intraperitoneal), as well as PCB mixtures (e.g., Aroclors 1254, 1221, and 1260) and individual PCB congeners (e.g., PCB 180 and 153). Outcomes reported in these studies include structural and morphological changes in various brain regions, alterations in calcium homeostasis, oxidative stress, neurotransmitter dynamics, apoptotic signaling, or changes in gene and protein expression. These findings highlight the broad neurotoxic potential of PCBs, with brain region and sex-specific differences that may underlie PCB-induced neurotoxic effects. Table A1, Table A2, Table A3 and Table A4 provide an overview of neurotoxic outcomes across exposure types and analyses.

#### 3.3.1. Histological/Morphological Changes

Structural alterations in the brain, including neuronal degeneration, tissue atrophy, and cell loss, are indicators of neurotoxic damage and neurodegeneration [102]. Only a few studies have investigated the effects of PCBs on histological or morphological endpoints in female rats. Female Sprague-Dawley rats exposed to inhaled Aroclor 1254 alone, or in combination with Aroclor 1221, did not show histopathological changes in the brain [24,25].

**Figure 4:**
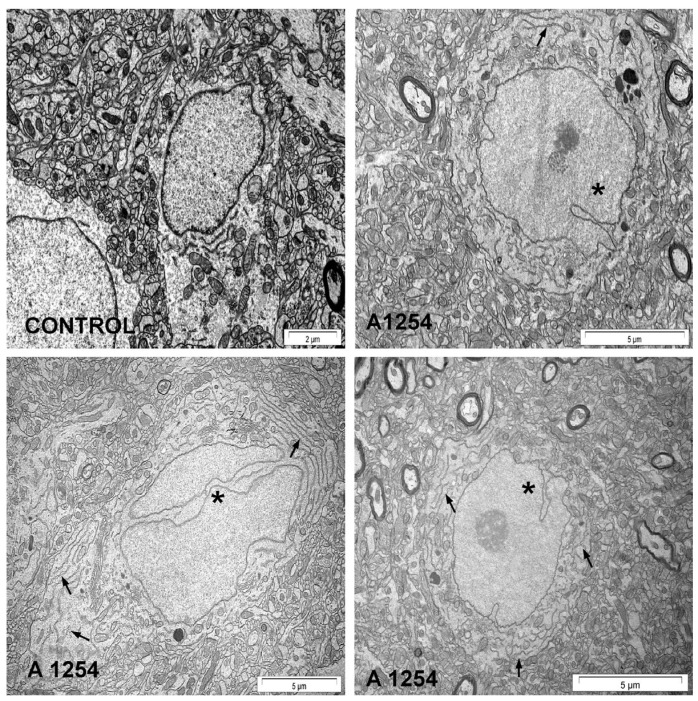
Electron micrograph showing the effect of PCBs on hippocampal sections in PCB-exposed adult male Wistar rats. (**Upper Left**) Control section. (**Upper Right**, **Lower Left**, **Lower Right**) PCB exposed sections. * Hypochromatic appearance of neurons and irregularly shaped and lobed cell nuclei. Arrows indicate enlargement and local edema of rough endoplasmic reticulum. Reprinted from [111], with permission from Elsevier.

In contrast, male Wistar rats exposed intraperitoneally (i.p.) to Aroclor 1254 exhibited significant histological and morphological changes across various brain regions [103,104,105,106,107,108,109,110]. In the cerebral cortex, neuronal shrinkage [104], degeneration [104,106], and the presence of pyknotic nuclei, accompanied by increased peri-neuronal spaces [103,104], were reported following PCB exposure. There was also an increase in pyknotic cell bodies, nonhomologous cytoplasm, and intense basophilic appearance in the cerebral cortex [107]. In contrast, pyramidal, Betz, and stellate cells, and myelin density were notably decreased in this region [107]. In the cerebellum, Purkinje neurons showed shrinkage [103,105], degeneration [103,105,106], and moderate atrophy [103]. The hippocampus displayed degeneration of pyramidal cells [103,108,109], neuronal shrinkage [108,109], and disruption in the Cornus Ammonis 4 region [110]. Male Wistar rats exposed to oral Aroclor 1254 showed a decreased number of neurons, and transmission electron microscopy identified irregularly shaped and lobed neuronal nuclei in the forebrain cortex and hippocampus, and enlargement and local edema of the rough endoplasmic reticulum in the hippocampus [111] (Figure 4).

#### 3.3.2. Calcium Modulation

Calcium homeostasis is essential for neuronal signaling, synaptic plasticity, and cell survival, with its disruption a hallmark of excitotoxicity and neurodegeneration [112]. Disruption of calcium signaling was observed across multiple brain regions in male rats exposed to Aroclor 1254, including alterations in calcium channel expression, kinase activity, and intracellular calcium handling [104,109,111,113,114]. Male Wistar rats exposed to i.p. Aroclor 1254 exhibited increased mRNA expression of *Cacna1d* in the cerebral cortex [104], but decreased mRNA expression of *Cacna1d* in the hippocampus [109]. PCB exposure decreased protein kinase A (PKA) alpha levels and increased protein expression of N-methyl-D-aspartate (NMDA) receptors, calpain, and N-type calcium channel protein alpha1B in the cerebral cortex [113]. In the hippocampus of male Wistar rats exposed to oral Aroclor 1254, PCBs decreased the maximum NMDA-induced calcium release [111]. Male Long-Evans rats exposed to Aroclor 1254 orally had reduced calcium uptake in the microsomes across the frontal cortex, cerebellum, and striatum [114]. Additionally, PCBs decreased calcium uptake in the mitochondria of the cerebellum and decreased total protein kinase C (PKC) activity while increasing membrane-bound PKC activity [114].

#### 3.3.3. Oxidative Stress and DNA Modifications

**Figure 5:**
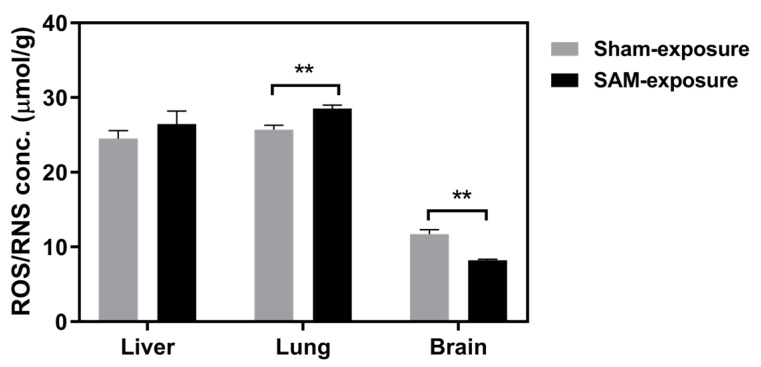
Biomarkers of oxidative stress (ROS/RNS) concentration in tissue homogenate after subchronic exposure to School Air Mixture (SAM, Aroclor 1254) vapor. Values are normalized by protein content and show mean ± standard error (*n* = 5 for the brain). ** *p* < 0.01, Student’s *t*-test, compared to sham exposure. Reprinted with permission from [24]. Copyright 2025 American Chemical Society.

Oxidative damage and impaired redox balance can compromise neuronal integrity, while alterations in DNA and antioxidant defenses can affect gene expression and neural resilience [115]. Female Sprague-Dawley rats exposed to inhaled Aroclor 1254 showed reduced reactive oxygen and nitrogen species (ROS/RNS) levels in the brain, whereas oxidized lipid markers, MDA and 4-HNE, were not altered following PCB inhalation (Figure 5) [24]. In a second study, female Sprague-Dawley rats exposed to an inhaled mixture of Aroclor 1254 and Aroclor 1221 did not have altered brain ROS/RNS levels [25].

Oxidative stress was observed in the brains of male rats following i.p. exposure to Aroclor 1254, characterized by elevated lipid and protein oxidation, disrupted expression of antioxidant enzymes, and increased DNA fragmentation across multiple brain regions [103,104,105,108,110,116,117]. Male Wistar rats exposed i.p. to Aroclor 1254 showed increased levels of lipid peroxidation [104,105,108,110,116,117,118], hydroxyl radicals [110,117,118], hydrogen peroxide [104,105,108,110,116,117], and protein carbonyls [104,105,108,116] in the cerebellum [105,117,118], cerebral cortex [104,117,118], and hippocampus [108,110,116,117,118]. PCB exposure decreased glutathione levels [117,118] and mRNA expression of copper/zinc superoxide dismutase (*Cu/Zn SOD*) [103] and *GPx-4* [103] in the cerebellum [103,117,118], cerebral cortex [103,117,118], and the hippocampus of male rats [103,117,118]. Furthermore, PCB exposure resulted in elevated levels of thiobarbituric acid reactive substances (TBARS) in the hippocampus [110] and resulted in DNA fragmentation [108]. PCB exposure led to a significant decrease in the levels of key antioxidant enzymes in the brain [103,110]. PCB exposure decreased mRNA expression of Cu/Zn SOD and glutathione peroxidase (GPx)-4 in the cerebellum, cerebral cortex, and hippocampus [103]. Additionally, PCBs decreased the concentrations of SOD, catalase (CAT), GPx, glutathione-S-transferase (GST), and glutathione reductase (GR) in the hippocampus [110]. Furthermore, PCB exposure lowered the specific activities of total SOD, Cu/Zn SOD, and manganese SOD (Mn SOD) across the cerebellum, cerebral cortex, and hippocampus, along with a decrease in the specific activity of GPx in these same regions [103]. Male and female Sprague-Dawley rats exposed to oral PCB 180 exhibited decreased glutathione levels in the cerebral cortex of males [119].

Oral exposure of male C57BL/6 mice to Aroclor 1254 induced significant oxidative stress in both the striatum and cerebellum [120]. PCB exposure increased the oxidized lipid markers MDA and 4-HNE in both regions, with greater accumulation observed in the striatum than in the cerebellum [120]. Additionally, PCB exposure led to an increase in the number of oxidized proteins in both the striatum and cerebellum. In response to this oxidative stress, PCB exposure also upregulated the protein expression of heme oxygenase-1 (HO-1) in both brain regions [120]. Furthermore, PCB exposure led to increased Mn SOD protein levels in the striatum and cerebellum, as well as an increase in Cu/Zn SOD protein expression in both regions [120].

#### 3.3.4. Neurotransmitters and Receptors

Disruptions in neurotransmitter systems and receptor signaling can impair synaptic transmission, cognitive function, mood regulation, and motor control [121]. The following sections examine how PCB exposure disrupts key neurotransmitter systems in rodent brains, including dopamine (DA) metabolism, and receptor/transporter expression; serotonin and norepinephrine dynamics; glutamatergic signaling and transporter regulation; and the biosynthesis machinery for neurotransmitters.

##### Biosynthesis of Neurotransmitters

**Figure 6:**
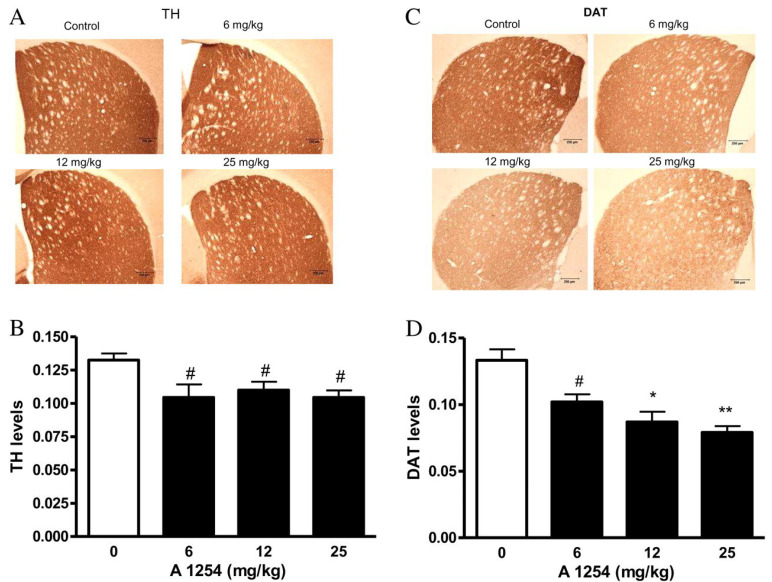
Tyrosine hydroxylase (TH) and dopamine transporter (DAT) and levels in the striatum of male C57BL/6 mice exposed orally to Aroclor 1254. (**A**) Thirty micrometer striatal sections were stained from mice 2 weeks after a 28-day exposure period for TH by immunohistochemistry. Scale bar represents 250 micrometers. (**B**) Staining levels were quantified by densitometry for TH. (**C**) Thirty micrometer striatal sections stained (immunohistochemistry) from mice 2 weeks after 28-day exposure. Scale bar represents 250 micrometers. (**D**) Staining levels quantified by densitometry for DA. # *p* < 0.05, * *p* < 0.01, and ** *p* < 0.001 from vehicle control [120] by permission of Oxford University Press.

Alterations in the biosynthesis of neurotransmitters in the brain can reduce the efficiency of neurotransmission and impair the function of neural networks [122]. Most studies investigating the effects of PCBs on neurotransmitter synthesis to date have focused on the expression and activity of tyrosine hydroxylase (TH), the rate-limiting enzyme of catecholamine biosynthesis [123]. Male Wistar rats exposed i.p. to Aroclor 1254 did not exhibit any changes in the mRNA expression of TH in the cerebellum or hippocampus. However, PCB exposure resulted in a decrease in protein expression of tyrosine hydroxylase in both the cerebellum and hippocampus [105,109]. Male Long-Evans rats exposed orally to Aroclor 1254 showed no changes in TH immunoreactivity or specific activity in the striatum [114]. Male C57BL/6 mice exposed orally to Aroclor 1254 exhibited a decrease in TH concentration in the striatum across all exposure groups (Figure 6A,B) [120].

##### Dopamine and Metabolites

DA plays critical roles in reward, motivation, and motor control; dysregulation is linked to neurological diseases [124,125]. Several studies have indicated PCBs influence levels of DA and its metabolites [126,127,128,129,130,131]. Consistent with these results, male Sprague-Dawley rats exposed orally to Aroclor 1254 exhibited varying effects on DA concentrations in striatal dialysate, as determined by a three-way analysis of variance (ANOVA) [132]. PCB exposure did not show a main effect on DA levels in the dialysate, but the interaction between PCB exposure and exposure duration was significant [132]. Specifically, PCB exposure increased DA concentrations after 3 days of exposure, but decreased DA concentrations after 1, 2, and 8 weeks [132]. In contrast, PCB exposure did not alter DA levels in the striatum [132]. Analogous studies have not been reported in female Sprague-Dawley rats. However, female Sprague-Dawley rats exposed to inhaled Aroclor 1254 and 1221 did not exhibit any alterations in DA or 3,4-dihydroxyphenylacetic acid (DOPAC) levels in the striatum [25].

Male Wistar rats exposed orally to Aroclor 1254 exhibited significant alterations in DA and its metabolites in specific brain regions [133]. PCB exposure resulted in a decrease in DA levels in the striatum, accompanied by reduced levels of its metabolites DOPAC and homovanillic acid (HVA) [133]. This exposure also resulted in a decrease in the DOPAC/DA and HVA/DA ratios in the striatum [133]. Similarly, while PCB exposure did not affect DA levels in the lateral olfactory tract, PCBs significantly reduced the levels of DOPAC, HVA, DOPAC/DA, and HVA/DA ratios in this region [133]. In a separate animal study, male Wistar rats exposed orally to Aroclor 1254 and 1260 showed significant changes in DA metabolism in the brain [134]. PCB exposure decreased DA and DOPAC concentrations in the caudate; however, PCB exposure did not affect HVA concentrations in either the caudate or the lateral olfactory tract, and DA and DOPAC levels remained unchanged in the lateral olfactory tract [134]. The DOPAC/DA ratio was decreased only in the lateral olfactory tract. At the same time, PCB exposure did not affect the HVA/DA ratios in either the caudate or lateral olfactory tract [134].

There is also evidence that PCB exposure alters brain DA homeostasis in male Long-Evans rats and male C57BL/6 mice. Analogous studies in the corresponding female animals have not been reported to date. Briefly, male Long-Evans rats exposed orally to Aroclor 1254 did not exhibit any changes in the levels of DA or HVA in the frontal cortex or striatum [114]. However, PCB exposure did lead to an increase in DOPAC levels in the striatum of exposed rats compared to controls [114]. Male C57BL/6 mice exposed orally to Aroclor 1254 exhibited an increase in DA levels; however, PCB exposure did not alter the levels of DOPAC, HVA, or the HVA/DA ratio in the striatum [120].

##### Dopamine Receptors and Transporters

Changes in dopamine receptor expression alter synaptic responsiveness and are implicated in neurodevelopmental and neuropsychiatric disorders [135]. Male and female Sprague-Dawley rats exposed orally to PCB 180 observed no alterations in specific binding to D1/D5 dopamine receptors in the cerebellum [119].

Male Wistar rats exposed i.p. to Aroclor 1254 exhibited significant changes in the expression of dopamine receptors in various brain regions [104,105,109]. PCB exposure increased the mRNA and protein expression of excitatory D1-like receptors (*DRD1* and *DRD5*) in the cerebellum while decreasing the mRNA and protein expression of inhibitory D2-like receptors (*DRD2*, *DRD3*, and *DRD4*) in this region [105]. Similarly, PCB exposure increased mRNA expression for D1-like receptors in the cerebral cortex, while reducing mRNA expression for D2-like receptors [104]. In the hippocampus, PCB exposure led to increased mRNA and protein expression of the excitatory D1-like receptors and decreased expression of the inhibitory D2-like receptors [109]. These studies in rats did not investigate changes in the expression of dopamine transporter (DAT). However, male C57BL/6 mice exposed orally to Aroclor 1254 showed a decrease in the concentration of DAT in the striatum [120] (Figure 6C,D).

##### Serotonin and Norepinephrine

Serotonin and norepinephrine (NE) are key modulators of mood, arousal, and stress response, where disruptions are associated with anxiety, depression, and cognitive deficits [136]. Several studies using synaptosomes suggest that PCB mixtures may affect serotonin homeostasis [128,131,137]. Moreover, the following evidence from rodent studies suggests alterations in serotonin metabolism in the brain following exposure to PCBs.

Male Wistar rats exposed orally to Aroclor 1254 and 1260 exhibited region-specific alterations in serotonin metabolism [138,139]. PCB exposure decreased serotonin levels in the frontal cortex and hippocampus, while increasing serotonin concentrations in the lateral olfactory tract; however, no changes were observed in serotonin concentrations in the brainstem or hypothalamus [139]. PCB exposure also increased 5-hydroxyindoleacetic acid (5-HIAA), the primary metabolite of serotonin, concentrations in the frontal cortex and lateral olfactory tract, but did not affect 5-HIAA concentrations in the hippocampus, brainstem, or hypothalamus [139]. The 5-HIAA/serotonin ratio was elevated in the frontal cortex, hippocampus, brainstem, and hypothalamus, though no changes were observed in the lateral olfactory tract [139]. Exposure to oral Aroclor 1254 and 1260 also showed region-specific changes in NE concentration. PCB exposure decreased NE concentrations in both the dorsal frontal cortex and hippocampus; however, PCBs did not affect NE concentrations in the medial-basal hypothalamus or brainstem [138].

Male Long-Evans rats exposed orally to Aroclor 1254 did not show any changes in the concentrations of NE or serotonin in the frontal cortex or striatum [114]. However, PCB exposure did increase the concentration of 5-HIAA in the striatum [114]. Male Wistar rats exposed orally to Aroclor 1254 did not show any changes in the concentration or activity of serotonin and NE in the striatum and lateral olfactory tract [133].

##### Glutamate

Glutamate is vital for synaptic plasticity as the primary excitatory neurotransmitter, where dysregulation contributes to excitotoxicity and neuronal injury [140]. Male Wistar rats exposed i.p. to Aroclor 1254 exhibited decreased mRNA expression of several key proteins relevant to glutamate synthesis and signaling in the cerebral cortex [113]. PCB exposure reduced the mRNA expression of the glial glutamate aspartate transporter (*GLAST*), brain-derived neurotrophic factor (*BDNF*), and neurotrophin tyrosine kinase receptor B (*NTRKB*) [113]. Additionally, PCB exposure decreased the mRNA expression of downstream signaling molecules, including microtubule-associated protein 1 (*MAP1)*, extracellular signal-regulated kinase 1 and 2 (*ERK1* and *ERK2)*, and the transcription factor cyclic AMP-response element binding protein (*CREB*) [113].

Male Wistar rats exposed orally to Aroclor 1254 exhibited several changes in glutamate uptake and transporter expression in freshly prepared whole-brain homogenates, synaptosomal fractions, and glial plasmalemmal vesicle (GPV) fractions. The rate of glutamate uptake into synaptosomes was increased, yet reduced in GPV fractions prepared from PCB-exposed male rats compared to the respective controls [141]. Additionally, the rate of glutamate accumulation was diminished at the point of maximal uptake in the GPV fraction [141]. PCB exposure also increased KCl-stimulated glutamate release in the synaptosomal fraction but did not alter the rate of released [^3^H] glutamate in the GPV fractions [141]. Furthermore, PCB exposure decreased glutamate transporter 1 (*GLT-1*) mRNA and protein expression in the forebrain, while *GLT-1* remained unchanged in the cerebellum [141]. Similarly, GLAST mRNA and protein expression were unchanged in both the forebrain and cerebellum [141]. PCB exposure increased the protein level of excitatory amino acid carrier 1 (EAAC1) in forebrain homogenates, although mRNA levels remained unchanged. In contrast, PCB exposure in cerebellar homogenates decreased *EAAC1* mRNA levels and showed a trend toward reduced protein levels [141].

Male rats exposed to oral Aroclor 1254 also exhibited significant alterations in NMDA-evoked glutamate signaling [111]. PCB exposure abolished NMDA-evoked glutamate release and reduced basal microdialysate glutamate concentration in hippocampal microdialysate [111]. The concentration of basal cyclic guanosine monophosphate (cGMP), a second messenger in glutamate neurotransmission, was not affected; however, PCB exposure decreased NMDA-evoked cGMP accumulation [111]. No studies investigated glutamate in female rats.

#### 3.3.5. Apoptosis

Apoptotic signaling in the brain, which plays a critical role in normal neuronal pruning during adolescent brain development [142], can also lead to programmed cell death in the adult brain, contributing to neurodegeneration and the loss of neural circuitry [143]. Male Wistar rats exposed i.p. to Aroclor 1254 exhibited significant changes in apoptosis-related signaling pathways in the cerebral cortex, cerebellum, and hippocampus [106,107,108]. PCB exposure increased nuclear factor kappa B (*NF-kB*) mRNA expression in both the cerebral cortex and cerebellum [106], as well as increased mRNA expression of pro-apoptotic proteins BH3 interacting-domain death agonist (*Bid*), BCL2-associated death promoter (*Bad*), BCL2-associated X protein (*Bax*), caspase 3 and 9 (*CASP3* and *CASP9*) in the cerebral cortex [106,107]. Additionally, PCB exposure decreased the mRNA expression of the anti-apoptotic protein B-cell leukemia/lymphoma 2 (*Bcl2*) in the cerebral cortex [106,107]. In the hippocampus, PCB exposure increased both mRNA and protein expression of pro-apoptotic genes *Bad*, *Bid*, and *Bax*, while decreasing the mRNA and protein expression of the anti-apoptotic gene *Bcl2* [108]. PCB exposure also led to increased mRNA and protein expression of pro-inflammatory markers, including tumor necrosis factor alpha (*TNFα*), *NF-kB*, tumor necrosis factor receptor superfamily member 6 (*Fas*), and Fas ligand (*FasL*) in the hippocampus [108], as well as increased *FasL* and caspase 8 (*CASP8*) mRNA expression in the cerebral cortex and cerebellum [106]. Furthermore, PCB exposure increased the mRNA and protein expression of *CASP3*, *CAP8*, and *CASP9* in the hippocampus, along with elevated caspase 3 activity in this region [108].

**Figure 7:**
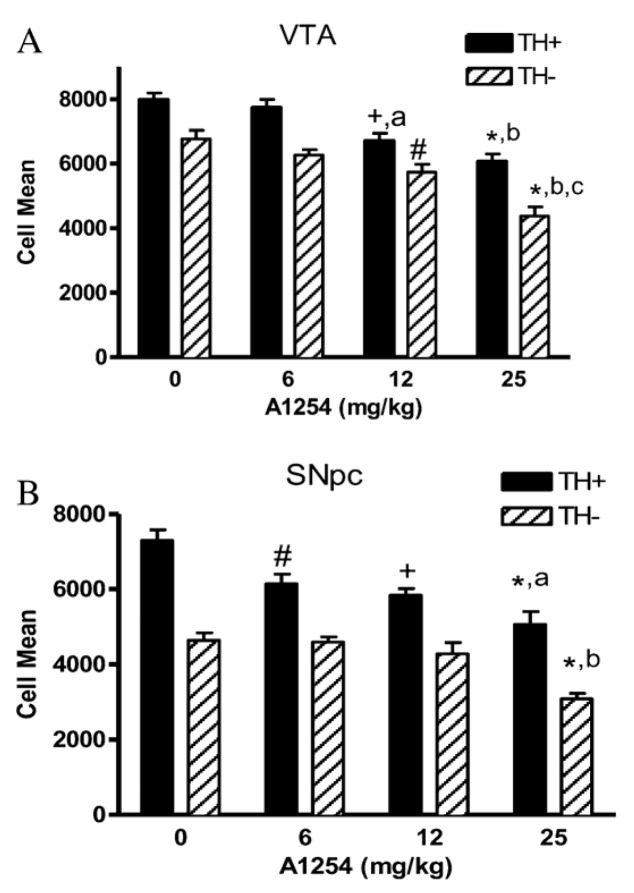
Effect of PCBs on neurons and tyrosine hydroxylase in the striatum of male C57BL/6 mice. TH-positive (TH+) and TH-negative (TH−) dopaminergic neurons counted from (**A**) VTA and (**B**) SNpc sections taken from mice 2 weeks following a 28-day exposure. Bars represent mean ± SEM, *n* = 7–8, # *p* < 0.01, + *p* < 0.001, and * *p* < 0.0001 from control; ^a^ *p* < 0.05 from 6 and 12 mg/kg A1254; ^b^ *p* < 0.005 from 6 and 12 mg/kg A1254; and ^c^ *p* < 0.001 from 12 mg/kg A1254 [120] by permission of Oxford University Press.

Male C57BL/6 mice exposed orally to Aroclor 1254 exhibited increased cell death in both TH-positive (TH+) and TH-negative (TH−) neurons in the substantia nigra pars compacta (SNpc) and ventral tegmental area (VTA) [120] (Figure 7).

#### 3.3.6. Additional Drivers of PCB-Induced Neurotoxicity

Additional molecular and cellular processes, including enzyme activities, receptor expression [144], iron homeostasis [145], amino acid levels [146], and gene expression have been investigated for their potential role in PCB neurotoxicity.

##### Enzymes

Changes in enzymatic activity can impact neurotransmitter metabolism, energy production, and ionic gradients, all of which are essential for normal neuronal function [147,148,149,150]. Male Sprague-Dawley rats exposed orally to Aroclor 1254 were evaluated for changes in nitric oxide synthase (NOS) activity using NADPH-diaphorase (NADPH-d) following hyperosmotic challenge. Hyperosmotic challenge involves i.p. injection of hypertonic saline and temporary water deprivation to induce intracellular dehydration and stimulate NOS activity in the brain. NOS-containing neurons were visualized using NADPH-d histochemistry, an established staining method in which reaction product intensity reflects NOS enzymatic activity due to the shared use of NADPH as a cofactor. Quantification was based on staining density in the supraoptic nucleus (SON). No significant difference in NADPH-d staining density in the SON was observed between hyperosmotic PCB-treated rats and hyperosmotic controls [151].

Male Wistar rats that were exposed to Aroclor 1254 via i.p. injection showed a reduction in the activity of membrane-bound ATPases [104,105,109,117], which are crucial for maintaining the ionic environment and signaling processes of neurons across brain regions [152,153,154,155]. Briefly, PCB exposure led to a reduction in the mean activity of Na^+^/K^+^ [104,105,109,117], Ca^2+^ [104,105,109,117], and Mg^2+^ ATPases [104,105,109,117] in the cerebral cortex [104,117], cerebellum [105,117], and hippocampus [109,117].

Creatine kinase plays a role in brain energetics and neurotransmitter trafficking between glial cells and neurons across multiple brain regions [156,157]. Male Wistar rats exposed i.p. to Aroclor 1254 showed a reduction in the activity of creatine kinase [104,105,118]. PCB exposure lowered the mean creatine kinase activity in the cerebral cortex [104,118], cerebellum [105,118], and hippocampus [118].

Acetylcholinesterase (AChE) is not only responsible for terminating synaptic transmission at cholinergic synapses but also has multifunctional, non-enzymatic roles in the brain that play a potential role in neurodegeneration [158,159]. Male Wistar rats exposed i.p. to Aroclor 1254 also exhibited a decrease in the activity of AChE [104,105,117]. PCB exposure reduced the mean AChE activity in the cerebellum [105,117], cerebral cortex [104,117], and hippocampus [117].

##### Receptors

Receptor dysregulation alters neuronal responsiveness and signaling pathways that influence cognition, behavior, and neuroendocrine balance [160,161,162]. Male and female Sprague-Dawley rats exposed i.p. to Aroclor 1221 exhibited sex-specific changes in the expression of several receptors in specific brain regions. In male rats, PCB exposure decreased mRNA expression of the androgen receptor and mu opioid receptor (*Oprm1*) in the preoptic area [163]. Additionally, PCB exposure increased mRNA expression of the *Oprm1* in the prefrontal cortex of male rats [163]. Notably, higher mRNA expression levels of the *Oprm1* in the prefrontal cortex were positively associated with increased time spent near the no-hormone stimulus animal during the sociosexual preference test, suggesting a mechanistic link between *Oprm1* expression and behavioral outcomes [163].

PCB exposure decreased the mRNA expression of vasopressin receptor 1a (*Avpr1a*) in the lateral septum of male rats exposed i.p. to Aroclor 1221 [163]. This study reported no changes in *Avpr1a* mRNA expression observed in the BNST, MeA, PVN, or VMH [163]. Male Wistar rats that were i.p. exposed to Aroclor 1254 exhibited reduced mRNA expression of estrogen receptors α and β in the hippocampus [116].

Female Sprague-Dawley rats exposed orally to PCB 153 exhibited changes in muscarinic receptor (MR) density across different brain regions. PCB exposure increased MR density in the cerebral cortex while decreasing MR density in the cerebellum; PCB exposure did not alter MR density in the hippocampus or striatum [164].

Male and female Sprague-Dawley rats exposed orally to PCB 180 did not show any alterations in high- or low-affinity sites on nicotinic receptors in the cerebellum [119].

##### Iron Metabolism

**Figure 8:**
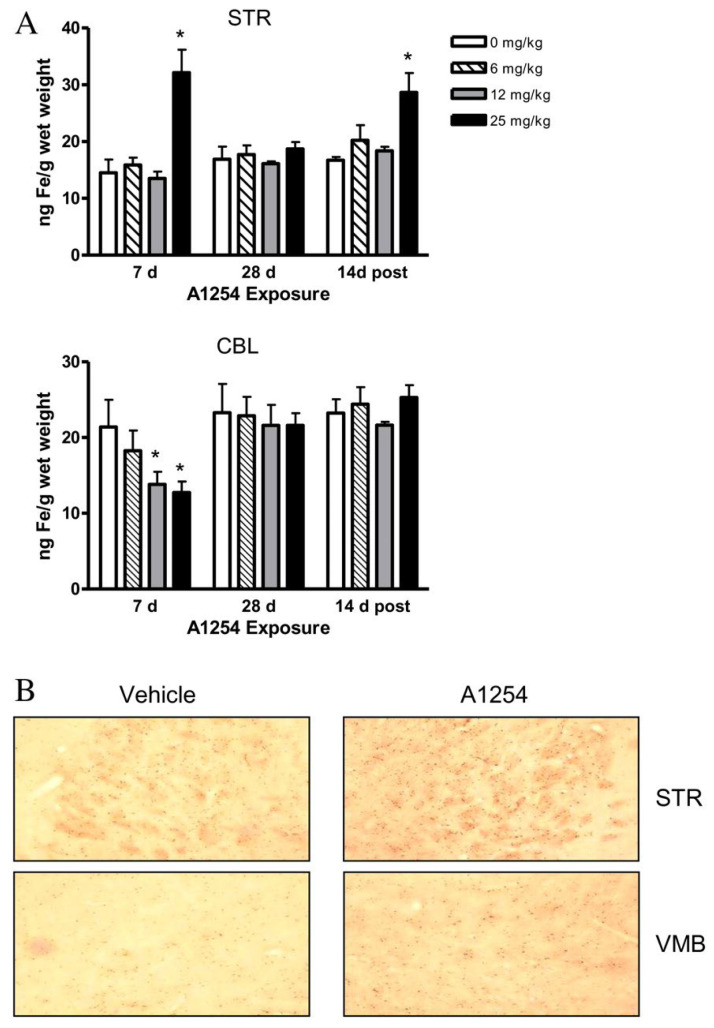
Effect of PCB exposure on iron in the brain of male C57BL/6 mice. (**A**) Striatal (STR) and cerebellar (CBL) tissue analyzed for total iron levels by atomic absorption spectroscopy (mean ± SEM; *n* = 4). * *p* < 0.05 from vehicle-control by ANOVA. (**B**) 30 µm sections of the striatum and midbrain (VMB) were processed for Perl’s staining and visualized microscopically. Representative brain sections (*n* = 3 per exposure group) are shown. Densitometric values were acquired using Image J software (mean ± SEM; *n* = 3). * *p* < 0.05 from vehicle control [120] by permission of Oxford University Press.

Iron accumulation in the brain can catalyze oxidative reactions and promote neurodegeneration [165,166]. Male C57BL/6 mice exposed orally to Aroclor 1254 exhibited significant changes in iron metabolism in the brain. PCB exposure elevated total iron (bound and unbound)in the striatum after 7 days of exposure and 2 weeks post-exposure, while exposure decreased total iron in the cerebellum at 7 days (Figure 8A) [120]. Additionally, PCB exposure increased the number of iron-positive cells in the striatum, but did not alter iron staining patterns in the cerebellum (Figure 8B) [120]. Further, PCB exposure increased ferritin levels in the striatum at 7 days of exposure, but ferritin levels decreased after 14 days of exposure, particularly in the 25 mg/kg group at 14 days post-exposure [120]. Finally, PCB exposure increased transferrin receptor 1 (TfR1) protein levels in the striatum [120].

##### Amino Acids

Amino acids serve as neurotransmitters and metabolic precursors; imbalances can impair neurotransmission and neuron-glia interactions [167,168,169]. Male and female Sprague-Dawley rats exposed to oral PCB 180 did not exhibit any changes in aspartate, glutamate, serine, glutamine, glycine, taurine, alanine, or γ-amino butyric acid in the cerebral cortex [119].

Male Wistar rats exposed orally to Aroclor 1254 exhibited reductions in basal levels of amino acids in hippocampal microdialysates. PCB exposure decreased the basal concentration of arginine [111]. However, PCB exposure did not alter the basal levels or NMDA-evoked release of taurine, nor did exposure alter aspartate accumulation, and no NMDA-evoked aspartate release observed [111].

##### Gene Expression

**Figure 9:**
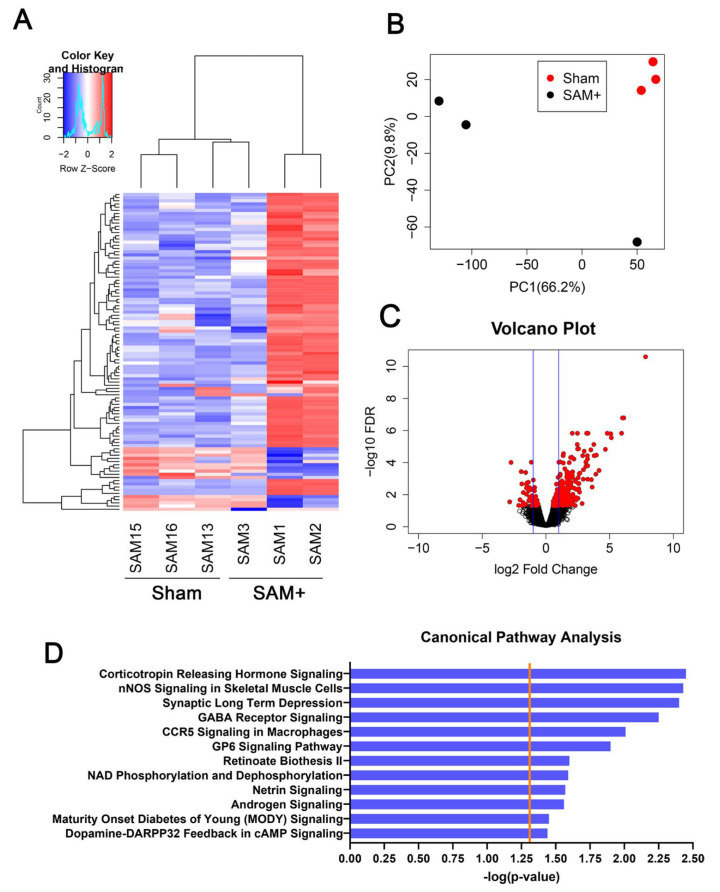
Effect of PCB exposure on gene expression in the brains of female Sprague-Dawley rats. RNA sequencing data visualization from brain tissues of School Air Mixture+ (SAM+, Aroclors 1245 and 1221) and sham (each *n* = 3). (**A**) Hierarchical clustering analysis and heatmap; each column represents an animal, and each row stands for a gene. (**B**) PCA plot displaying all six samples along PC1 and PC2. (**C**) Volcano plot visualizing the log2 fold change against the -log 10 false discovery rate (FDR). Dots in red indicate FDR < 0.05, dots in black indicate FDR ≥ 0.05, and blue vertical lines represent a fold change of 2. (**D**) Ingenuity pathway analysis (IPA)-identified statistically significant canonical pathways *(p* < 0.05) for the differentially expressed genes. The orange vertical line represents the threshold p-value of 0.05. Reprinted with permission from [25]. Copyright 2025 American Chemical Society.

Gene expression changes reveal shifts in molecular signaling that may underlie structural or functional brain alterations [170,171,172]. Female Sprague-Dawley rats exposed to inhaled Aroclor 1254 and 1221 exhibited changes in differentially expressed genes (DEGs) and alterations in canonical pathways in the brain [25]. In the whole brain, PCB exposure resulted in 274 upregulated genes and 58 downregulated genes, as well as pathways related to neurotransmitter signaling, cognitive dysfunction, vascular function, and immune response (Figure 9) [25].

Male Wistar rats i.p. exposed to Aroclor 1254 exhibited significant changes in the expression of tight junctional proteins in the hippocampus. PCB exposure led to a decrease in mRNA expression of integral membrane proteins, including Occludin (*Ocln*) and Claudin-5 (*Cldn5*), as well as cytoplasmic accessory proteins Zona Occludens-1 and -2 (*ZO-1* and *ZO-2*), Afadin (*AF-6*), and the Junction Adhesion Molecule-3 (*Jam3*) [116].

Male Wistar rats i.p. exposed to Aroclor 1254 exhibited decreased mRNA and protein expression of the cytoskeletal neurofilament light chain (*Nefl)* in neuronal cells of the cerebral cortex and cerebellum [106].

##### Signaling Pathways

Disruption of intracellular signaling cascades, such as MAPK/ERK and cGMP, affects synaptic plasticity, learning, and neuronal survival [173,174]. Male Wistar rats exposed orally to Aroclor 1254 exhibited a disruption in NMDA receptor–dependent intracellular signaling. While basal cGMP levels were unaffected, PCB exposure significantly reduced NMDA-evoked cGMP accumulation [111].

Male Wistar rats i.p. exposed to Aroclor 1254 exhibited significant changes in the expression of proteins involved in growth-stimulatory signaling pathways in the hippocampus. PCB exposure resulted in a significant reduction in expression of the neurotrophic ligand BDNF, and the BDNF receptor NTRKB [116]. Additionally, PCB exposure suppressed components of neurotrophin-associated signal transduction pathways, including the Ras, Raf, and MAPK/ERK signaling cascade [116].

##### Cytokine/Chemokine

Neuroinflammation driven by cytokines and chemokines contributes to blood–brain barrier disruption, glial activation, and neuronal injury [175,176]. Female Sprague-Dawley rats exposed to inhaled Aroclor 1254 and 1221 did not exhibit altered cytokine or chemokine concentrations in whole brain homogenate [25].

##### DNA Methylation

Epigenetic modifications such as DNA methylation regulate gene expression in the brain and may mediate long-term effects of toxic exposures on neural development and behavior [177,178,179]. Male and female Sprague-Dawley rats exposed i.p.to Aroclor 1221 did not show any alterations in DNA methylation in the bed nucleus of the stria terminalis (BNST), medial amygdala (MeA), paraventricular nucleus (PVN), or ventromedial hypothalamus (VMH) [163].

### 3.4. Behavioral Outcomes

This section summarizes the effects of PCB exposure on behavioral outcomes in rodent models, based on data from studies using various PCB mixtures (e.g., Aroclors 1221, 1248, 1254) and congeners (e.g., PCB 77, PCB 180), exposure routes (oral, i.p., inhalation, subcutaneous), and timepoints (acute, subchronic, chronic). Of the 15 studies reporting neurotoxic outcomes, 6 studies examined only males, 4 investigated males and females, and 5 studies examined only female rodents. Behavioral domains assessed include locomotion, exploration, anxiety-like responses, learning and memory, motor coordination, social and sociosexual behavior, operant conditioning, maternal behavior, cognitive flexibility, and impulsivity. Both male and female rats and mice were studied, although several studies focused on males alone. The results reveal PCB-induced behavioral alterations that are exposure-dependent and often sex-specific, with some effects emerging only after repeated testing or in particular test phases. Table A5, Table A6 and Table A7 provide an overview of behavioral outcomes across exposure types and test paradigms.

#### 3.4.1. Locomotor and Exploration

Locomotor and exploratory behaviors are commonly assessed to detect disruptions in central nervous system (CNS) function, including alterations in arousal, motivation, and sensorimotor processing [180]. These behaviors are typically evaluated using the Open Field Test (OFT), which measures spontaneous movement in a novel arena and quantifies metrics such as distance traveled, zone preference (inner vs. outer), and habituation over repeated trials [181]. Alterations in locomotor activity or exploration patterns may indicate neurotoxic effects on dopaminergic or serotonergic systems, as well as impairments in habituation or anxiety regulation [182]. Changes in horizontal and vertical activity levels can further reflect disruptions in motor coordination or motivation resulting from neurotoxic insult [183].

Male and female Sprague-Dawley rats orally exposed to PCB 180 showed changes in locomotion and exploration [119]. During the OFT, performed 24 days after exposure (first test day), females exhibited a significant dose-dependent increase in the percentage of time and distance traveled in the inner zone of the open field, while the percentage of time and distance in the outer zone of the open field decreased [119]. These behavioral differences diminished over five days of repeated testing, with significant interactions between exposure and test day for both time and distance in the inner zone. Habituation metrics, calculated as the ratio of behavior across days 2–5 to day 1, revealed dose-dependent impairments for time and distance [119]. The total distance moved across both zones exhibited a quadratic dose–response pattern, with increased activity at intermediate doses but no consistent changes over time. In contrast, no significant behavioral changes were detected in males exposed to PCB 180 at any exposure level [119].

Male Wistar rats i.p. exposed to Aroclor 1254 exhibited changes in locomotion and exploration [107,110]. One study reported decreased exploratory behavior, number of crossings in peripheral and center squares, and number of grooming in rats exposed to PCBs in the OFT [107]. Rats experienced an increased number of fecal boli, grooming, rearing, and freeze bouts compared to control animals [107]. However, a second study reported increased exploratory behavior, locomotion in all squares of the field, and time spent in outer squares in male rats after PCB exposure in the OFT [110]. They also reported no change in the number of fecal boli, grooming, and freeze bouts compared to control animals [110]. Although both studies assessed locomotor and exploratory behaviors in the OFT after 30 days of i.p. exposure to 2 mg/kg/day Aroclor 1254 in corn oil, methodological differences may explain the contrasting results. Specifically, variations in the testing apparatus (arena size, wall height, and floor color), lighting, and starting position may influence anxiety-like and exploratory behaviors, potentially contributing to the increased center avoidance and fecal boli in the first study versus the increased locomotion and exploration in the second.

Male Long-Evans rats exposed to oral Aroclor 1245 at varying doses exhibited changes in locomotion and exploration. Rats repeatedly exposed to Aroclor 1254 at 30 mg/kg demonstrated significantly reduced horizontal motor activity compared to both control and 10 mg/kg groups; however, vertical activity was unaffected by PCB exposure [114]. Rats acutely exposed to Aroclor 1254 exhibited dose-dependent effects on motor activity. Briefly, exposure to 1000 mg/kg resulted in significantly decreased horizontal motor activity at both 45 min and 7 days after exposure [184]. Vertical activity was significantly reduced at 300 mg/kg and above, 45 min post-dosing. No significant effects were observed at 100 mg/kg [184]. Repeated administration of Aroclor 1254 resulted in significant reductions in horizontal motor activity on days 7 and 14 at 100 mg/kg, days 14 to 42 at 30 mg/kg, and day 22 at 10 mg/kg, with full recovery observed by day 49 at 10 and 30 mg/kg [184]. Vertical activity was significantly reduced from day 22 to 42 at 30 mg/kg and on days 7 and 14 at 100 mg/kg, while no vertical activity changes were observed at 10 mg/kg. An additional follow-up study confirmed no changes in activity at doses ≤10 mg/kg given for six weeks [184].

**Figure 10:**
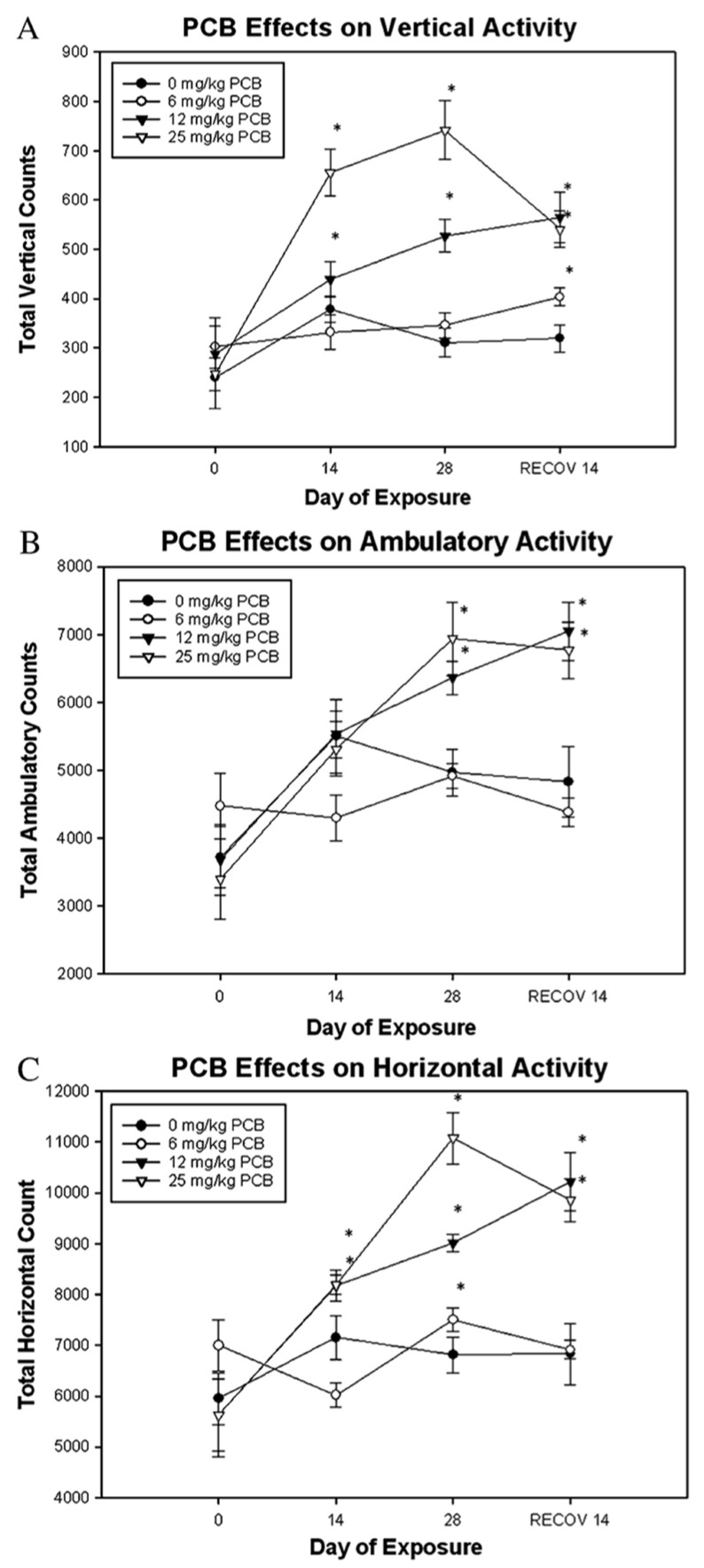
Effect of PCBs on activity in open field test in male C57BL/6 mice. (**A**) Total vertical, (**B**) ambulatory, and (**C**) horizontal locomotor activities were measured in mice exposed to vehicle or 6–25 mg/kg A1254. Data represents total activity counts ± SEM (*n* = 10) at week 0 (baseline) and at the end of exposure weeks 2, 4, and 2 weeks postexposure. * Denotes significant differences (*p* < 0.0001) from vehicle control at the same time point by repeated measures ANOVA [120] by permission of Oxford University Press.

Male C57BL/6 mice orally exposed for 4 weeks to Aroclor 1245 doses also exhibited changes in locomotion and exploration; however, these effects differ from those observed in rats described above. Briefly, mice exposed to 12 and 25 mg/kg Aroclor 1254 showed elevated vertical activity after 2 weeks, which further increased by week 4 and remained elevated for at least 2 weeks post-exposure [120] (Figure 10). Similarly, these higher dose groups exhibited increases in ambulatory activity by the end of the 4-week exposure period, which continued after cessation of exposure [120]. While mice exposed to 6 mg/kg showed decreased ambulation at week 2, their activity did not differ significantly from that of controls over time. Horizontal activity was also significantly increased in mice exposed to 12 and 25 mg/kg Aroclor 1254 starting at week 2 and remained elevated through the post-exposure period [120].

#### 3.4.2. Anxiety-like Behavior

Anxiety-like behavior is frequently assessed in rodents to detect changes in CNS function related to emotional regulation, risk assessment, and behavioral inhibition [185]. The Elevated Plus Maze (EPM) relies on rodents’ natural aversion to open, elevated spaces and preference for enclosed arms to infer anxiety levels [186,187]. In the EPM test, metrics such as the time spent in open versus closed arms, the number of open arm entries, and the total arm entries are quantified to evaluate anxiety-related behavior and general activity [188,189]. Alterations in EPM performance may reflect neurotoxic impacts on GABAergic, serotonergic, or glutamatergic signaling pathways, which are critical in the modulation of anxiety and stress responses [185,187]. Changes in open arm avoidance can indicate heightened anxiety or impaired behavioral inhibition due to neurotoxic exposures [186]. Similarly, the light-dark box test [190] utilizes rodents’ natural aversion to brightly lit environments, with increased time spent in the dark compartment and fewer transitions reflecting elevated anxiety [191]. This test is sensitive to pharmacological and toxicological manipulations of anxiety-regulating pathways and complements EPM findings in behavioral phenotyping [191].

**Figure 11:**
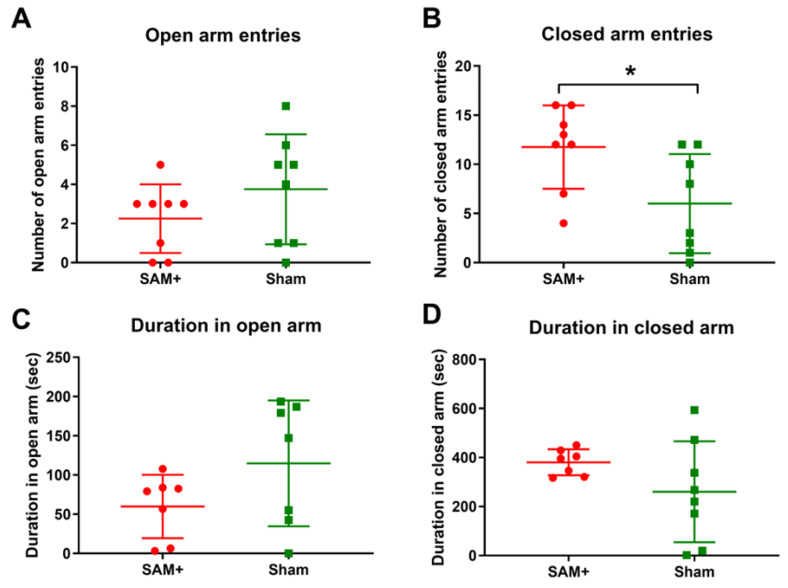
Effect of PCBs on elevated plus maze performance in female Sprague-Dawley rats exposed to School Air Mixture+ (SAM+, Aroclors 1245 and 1221). (**A**,**B**) show open- and closed-arm entries, respectively. (**C**,**D**) shows the time spent in open and closed arms. Values are mean ± SE (*n* = 8). * *p* < 0.05, Student’s *t*-test. Reprinted with permission from [25]. Copyright 2025 American Chemical Society.

Male and female Sprague-Dawley rats exposed to i.p. Aroclor 1221 did not exhibit changes in anxiety-like behavior [192]. In the EPM, there was no change in time spent in the arms or the number of entries into open/closed arms during juvenile or adult periods [192]. During the light-dark box test, there was no change in time or number of entries in the light side of the box [192]. In contrast, female Sprague-Dawley rats exposed to a mixture of inhaled Aroclor 1221 and 1254 showed changes in anxiety-like behavior. In the EPM test, the female rats exhibited an increase in the duration and number of entries in the closed arm [25] (Figure 11). Male Wistar rats exposed to i.p. Aroclor 1254 also exhibited changes in the EPM. Similarly to the inhalation study, there was a significant increase in closed arm entries, while the time spent in the open and closed arms was significantly decreased [107].

#### 3.4.3. Learning and Memory

Learning and memory behaviors are widely assessed to evaluate the integrity of cognitive function and identify disruptions in processes such as spatial navigation, working memory, and associative learning [193,194]. These are commonly examined using well-established behavioral tests, including the Morris water maze (MWM) [195], the eight-arm radial maze [196], and the conditioned flavor aversion (CFA) test [197]. The MWM assesses spatial learning and memory by requiring animals to locate a hidden platform in a pool of water using distal visual cues, providing quantitative measures such as escape latency, path length, and time spent in the target quadrant during probe trials [198,199]. The 8-arm radial maze evaluates working and reference memory by tracking arm entries and re-entries during baited trials, revealing deficits in short-term memory retention or spatial strategy use [200]. CFA tests associative learning by pairing a novel flavor with an aversive stimulus, measuring subsequent avoidance behavior to detect impairments in conditioned learning [201]. Alterations in performance across these tasks can reflect neurotoxic effects on hippocampal, cortical, or limbic system circuits critical for memory encoding, retrieval, and aversive learning [202,203,204].

**Figure 12:**
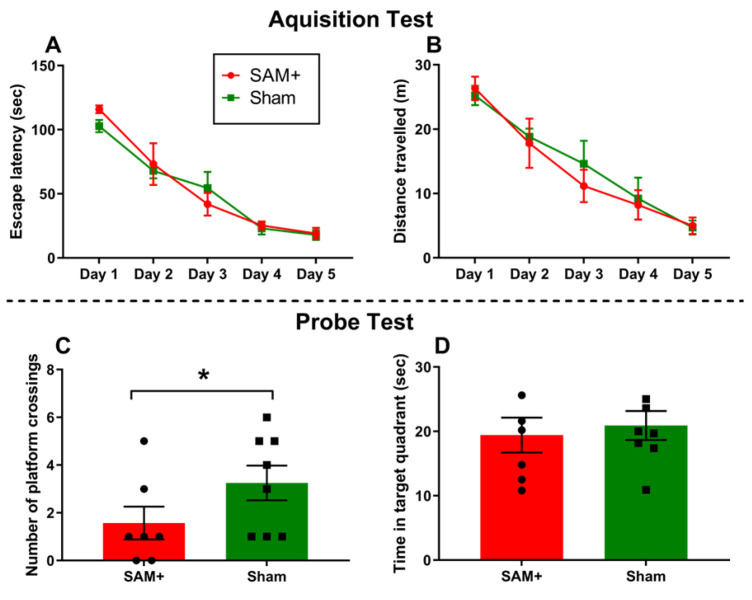
Effect of PCBs on Morris water maze performance in female Sprague-Dawley rats exposed to School Air Mixture+ (SAM+, Aroclors 1245 and 1221). The five-day acquisition test is illustrated in (**A**), showing escape latency, and (**B**) showing swimming distance traveled. Data from day 6 probe test shows (**C**), the number of platform crossings, and (**D**), the time spent in the target quadrant. Values are mean ± SE (*n* = 7 for SAM+, *n* = 8 for sham). * *p* < 0.05, Student’s *t*-test. Reprinted with permission from [25]. Copyright 2025 American Chemical Society.

**Figure 13:**
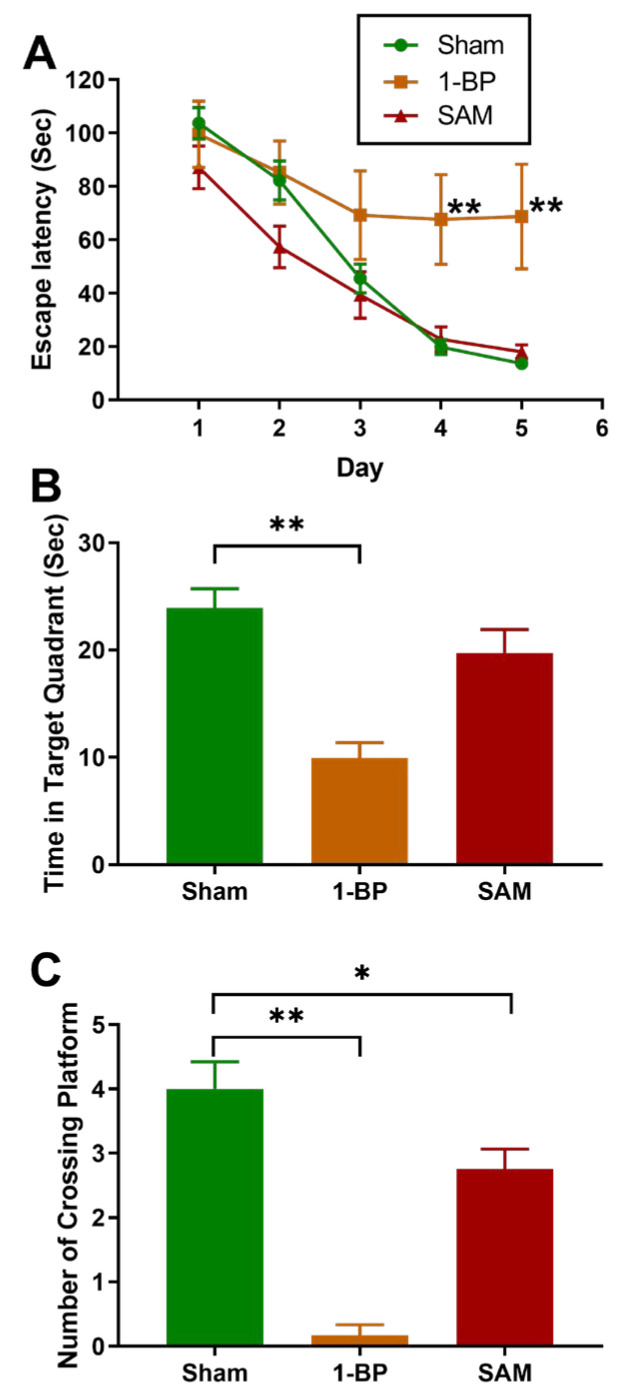
Effect of PCBs on Morris water maze performance in female Sprague-Dawley rats exposed to School Air Mixture (SAM, Aroclor 1245) or 1-bromopropane (1-BP, positive control). Five-day acquisition test (**A**) showing escape latency. Data from day 6 probe test showing (**B**), time spent in the target quadrant and (**C**), number of platform crossings. Data expressed as mean ± standard error (*n* = 8 for SAM and 1-BP, *n* = 6 for sham). * *p* < 0.05, ** *p* < 0.01, vs. sham group. (**A**) Two-way repeated-measures ANOVA. (**B**,**C**) One-way ANOVA with Bonferroni correction for multiple comparisons. Reprinted with permission from [24]. Copyright 2025 American Chemical Society.

Female Sprague-Dawley rats exposed to inhaled Aroclor 1254 alone, or in combination with Aroclor 1221, showed changes in the MWM. There was no significant difference in escape latency, travel distance, or time spent in the target quadrant in PCB-exposed rats; however, PCB-exposed rats exhibited fewer platform crossings than controls [24,25] (Figure 12 and Figure 13).

Male Wistar rats i.p. exposed to Aroclor 1254 exhibited changes in learning and memory with the 8-arm radial maze [110]. In the total memory error (TME) analysis, PCB-exposed rats showed an initial decline in TME, followed by inconsistent performance with no sustained improvement in later trials [110]. In the long-term memory error (LTME) analysis, PCB-exposed rats showed an increase in LTME over time [110]. Rats exposed to PCBs exhibited erratic performance with no apparent reduction in short-term memory errors (STME) throughout the experiment [110].

Male Long-Evans rats exposed to various doses of oral Aroclor 1245 exhibited changes in the flavor aversion test. Following acute exposure, rats had reduced saccharin preference at doses ≥25 mg/kg compared to vehicle-treated controls [184]. The reduction in saccharin intake occurred independently of total fluid intake, which was significantly decreased only at the 15 mg/kg dose. Repeated exposure to Aroclor 1254 also showed aversive effects on flavor preference [184]. In the first conditioning experiment, repeated pairing of saccharin with 15 mg/kg Aroclor 1254 significantly decreased daily saccharin intake; however, the 7.5 mg/kg dose had no effect when saccharin was the sole fluid available. In the following choice test conducted after 14 pairings, rats treated with 15 mg/kg Aroclor 1254 demonstrated a reduction in saccharin preference. A follow-up experiment using repeated doses of 3.75 and 7.5 mg/kg Aroclor 1254 over 14 days revealed no significant effects on saccharin intake, either during conditioning or in subsequent choice testing [184]. Total fluid intake during the choice test was also reduced following repeated exposure to 15 mg/kg Aroclor 1254 [184].

#### 3.4.4. Motor Coordination and Balance

Motor coordination and balance are key components of neuromotor function and are frequently evaluated to detect impairments in cerebellar or sensorimotor integrity following toxicant exposure or neurological insult [205,206]. These are commonly assessed using well-established behavioral assays, such as the accelerating rotarod test, which provides sensitive measures of motor performance and motor learning [207,208]. In this task, rodents are placed on a rotating rod that gradually accelerates, and latency to fall is recorded across repeated trials [209]. This test evaluates a combination of balance, coordination, grip strength, and motor learning, with decreased performance indicating deficits in neuromuscular control or cerebellar function [210]. Alterations in rotarod performance can reflect disruptions in central motor pathways, including cerebellar Purkinje cell function, basal ganglia signaling, or corticospinal tract integrity [211,212,213].

Male Wistar rats exposed to i.p. Aroclor 1254 exhibited differences in motor coordination in the rotarod test. PCB-exposed rats did not stay on the rotating rod as long as controls and had an increased number of falls compared with controls [107].

#### 3.4.5. Social and Sociosexual Behavior

Social and sociosexual behaviors are commonly assessed to evaluate neural circuits responsible for social motivation, recognition, and affiliative communication [214]. These behaviors are typically examined using tests such as the three-chamber social interaction test, which measures preference for a chamber containing an animal of the same species versus an empty chamber [215], or the social novelty test, which distinguishes recognition and preference for familiar versus novel stimulus animals [216]. Outcomes such as latency to approach, time spent in proximity to the stimulus animal, and number of transitions between chambers provide insight into social engagement and discrimination [217]. Sociosexual behaviors may also be assessed through ultrasonic vocalizations (USVs), with specific call types reflecting affiliative or sexually motivated communication [218]. Alterations in social investigation, recognition, or sociosexual USVs can signal disruptions in neural systems involved in social reward processing, olfactory signaling, or anxiety regulation, potentially indicating neurodevelopmental or neurotoxic effects [218,219].

Male and female Sprague-Dawley rats exposed to Aroclor 1221 i.p. showed differences in social and sociosexual behavior. In the first stage of the social approach test, where juvenile mice were given the choice between a chamber containing a stimulus animal and an empty chamber, no significant effects of PCB exposure were observed on the total time spent in the area near the stimulus animal [192]. However, juvenile PCB-exposed females exhibited a significantly increased latency to approach the area near the stimulus animal. No effect was observed in males [192]. In the second stage of the test, assessing social recognition and preference by offering a choice between a familiar and a novel stimulus animal, PCB exposure did not influence the time spent near, or latency to reach, either the familiar or novel animal [192]. PCB exposure did not alter sociosexual ultrasonic vocalizations, but sociosexual choice was altered. In males, PCB exposure significantly increased the time spent near a no-hormone (non-estrous) female stimulus [192]. While the time spent near the hormone-treated female was not significantly altered, the combined increase in time spent near both stimulus animals resulted in an overall increase in total interaction time among juvenile-exposed males [192]. PCB exposure did not alter affiliate USVs, but affiliative behavior was changed in females with an increased latency to hop [192].

#### 3.4.6. Operant Conditioning and Behavioral Flexibility

Operant conditioning paradigms are widely used to assess learning, motivation, and behavioral flexibility, providing insight into the reward-related and executive function pathways in the CNS [220,221]. These tasks, including fixed interval (FI), extinction (EXT), and transition schedule testing, allow for the quantification of response patterns such as lever pressing rates, response bursts, and extinction persistence [222,223]. Alterations in operant performance can reflect disruptions in dopaminergic and corticostriatal circuitry, which are critical for timing, reinforcement processing, and behavioral inhibition [224,225]. Changes in response trajectories across FIs or increased responding during EXT phases may indicate neurotoxic effects on impulse control or motivational drive [226,227]. Additionally, heightened response rates during transitions between reinforcement schedules may signal impaired adaptability and behavioral regulation, often linked to prefrontal or striatal dysfunction [228,229]. Therefore, these patterns are valuable in identifying neurobehavioral toxicity following exposure to environmental contaminants, such as PCBs.

Male Sprague-Dawley rats exposed orally to Aroclor 1248 exhibited alterations in operant training tests [230]. Rats exposed to PCB significantly increased lever pressing rate during FI sessions compared to controls. A mixed ANOVA revealed a main effect of segment duration, with lever presses increasing progressively across the 30 s segments [230]. Follow-up analysis revealed that the PCB and control groups exhibited distinct response trajectories over the 120 s interval. Specifically, the PCB group showed significantly greater lever pressing than controls by 90 s and 120 s, but not at earlier time points. During the last six sessions, there was no change in response bursts, and no significant differences were observed in the number of reinforcements delivered across groups during the final six sessions [230].

During EXT testing in male rats, the PCB-food group exhibited an increase in responding across the 5 min EXT period, in contrast to the relatively steady pattern observed in controls. No significant differences were detected between the PCB and control groups in extinction response patterns [230]. During transition training testing, male rats exposed to PCB exhibited altered behavioral responses; there was an increase in lever pressing during the first session of a new schedule compared to the second in PCB-exposed rats. Specifically, during continuous reinforcement (CRF) and FI schedules, the PCB group demonstrated a significant main effect of schedule session, with higher response counts in earlier sessions. PCB-exposed rats displayed more response bursts than control animals [230].

During EXT components associated with transition schedules, the PCB-exposed male rats pressed significantly more than the control rats. Across FI schedules, PCB-exposed rats exhibited the greatest number of extinction presses during FI 30 s and progressively fewer across the 06- and 120 s reinforcement conditions [230]. A significant three-way interaction (Groups × Associated Schedule × Schedule Session) confirmed that the pattern of extinction responding across sessions was uniquely altered in the PCB group compared to controls [230].

Male and female Sprague-Dawley rats exposed to inhaled Aroclor 1248 exhibited changes in operant training tests. PCB-exposed rats showed significantly higher response rates during the final stable-state sessions of the 120 s FI, 5 min EXT schedule [231]. A mixed ANOVA revealed a main effect of group and segment, as well as a significant interaction between group and segment. Follow-up comparisons showed that the PCB group exhibited elevated responding specifically at 90 and 120 s into the FI interval, with no significant differences from controls at the 30 or 60 s segments [231]. Male rats exposed to PCBs responded significantly more than male controls, while female rats in the PCB group did not differ from female controls. Additionally, males in the PCB group pressed significantly more than females. During the final two stable-state sessions, response bursts were more frequent in the PCB group compared to controls [231].

#### 3.4.7. Reproductive and Maternal Behavior

Reproductive and maternal behaviors are frequently assessed in rodent models to identify alterations in neuroendocrine and motivational systems that regulate social, sexual, and caregiving behaviors [232]. Female sexual behavior is commonly evaluated using lordosis and pacing paradigms, which measure receptivity, sexual motivation, and control over mating interactions based on latency to approach, return after mounts or intromissions, and lordosis response [233]. Maternal behavior is a sensitive indicator of CNS integrity and hormonal regulation and is typically assessed through pup-directed activities such as licking/grooming, nursing posture, nest-building, and pup retrieval [234,235]. Changes in these behaviors may reflect alterations in hypothalamic-pituitary-gonadal or -adrenal axis signaling, dopaminergic systems, or disruptions in circuits responsible for social motivation [236,237].

Adult female Long-Evans rats exposed to i.p. Aroclor 1221 and 1254 during lactation did not show changes in reproductive behavior [238]. Briefly, female sexual behavior was assessed using traditional lordosis tests to measure receptivity and pacing tests that evaluated sexual motivation and control through latency and escape behaviors in a two-compartment chamber [238]. There were no changes observed in approach latency, mount return latency, intromission return latency, post-ejaculatory refractory period, or lordosis quotient for pacing and non-pacing [238].

In a separate study, female Long-Evans rats exposed to PCB 77 subcutaneously from gestational days 6-18 exhibited changes in maternal behavior. PCB exposure significantly increased the amount of time dams spent licking and grooming their pups, as well as the number of nursing bouts and the time spent on the nest [239]. High crouch nursing posture, associated with attentive maternal care, was significantly reduced in PCB-exposed rats [239]. However, PCB exposure did not change the time spent engaged in maternal autogrooming, or the time spent nursing pups [239].

Female Swiss Albino mice exposed orally to a mixture of six non-dioxin-like PCBs, including PCBs 28, 52, 101, 138, 153, and 180, did not show changes in maternal behavior. Briefly, there was no change in nest-building activity or the time to retrieve a pup during the nursing period after PCB exposure [240].

#### 3.4.8. Cognitive Flexibility and Decision-Making

Cognitive flexibility and decision-making are commonly assessed using operant-based paradigms that test an animal’s ability to modify behavioral strategies in response to changing rules and reinforcement contingencies [222]. These tasks assess the functional integrity of prefrontal and striatal circuits involved in attentional control, behavioral inhibition, and adaptive learning [241]. One assessment is the set-shifting and reversal learning task, which consists of three sequential phases. In the visual cue discrimination phase, animals learn to associate a visual stimulus (an illuminated cue light) with a food reward, requiring attention to external cues [242]. This is followed by the set-shifting (position discrimination) phase, in which the rule shifts to a fixed spatial location, testing the animal’s ability to inhibit the prior rule and adopt a new response strategy [242]. The final reversal learning phase requires animals to reverse the spatial contingency, thereby assessing behavioral flexibility and the capacity for response inhibition [242]. Performance metrics such as total errors to criterion, trial omissions, response latency, and error subtypes (perseverative or regressive errors) provide a characterization of learning strategies and potential deficits in executive function [243]. Therefore, assessments of cognitive flexibility and decision-making provide a means of detecting disruptions in executive function associated with PCB exposure, shedding light on the potential neurobehavioral effects of environmental pollutants on the prefrontal-striatal circuitry.

**Figure 14:**
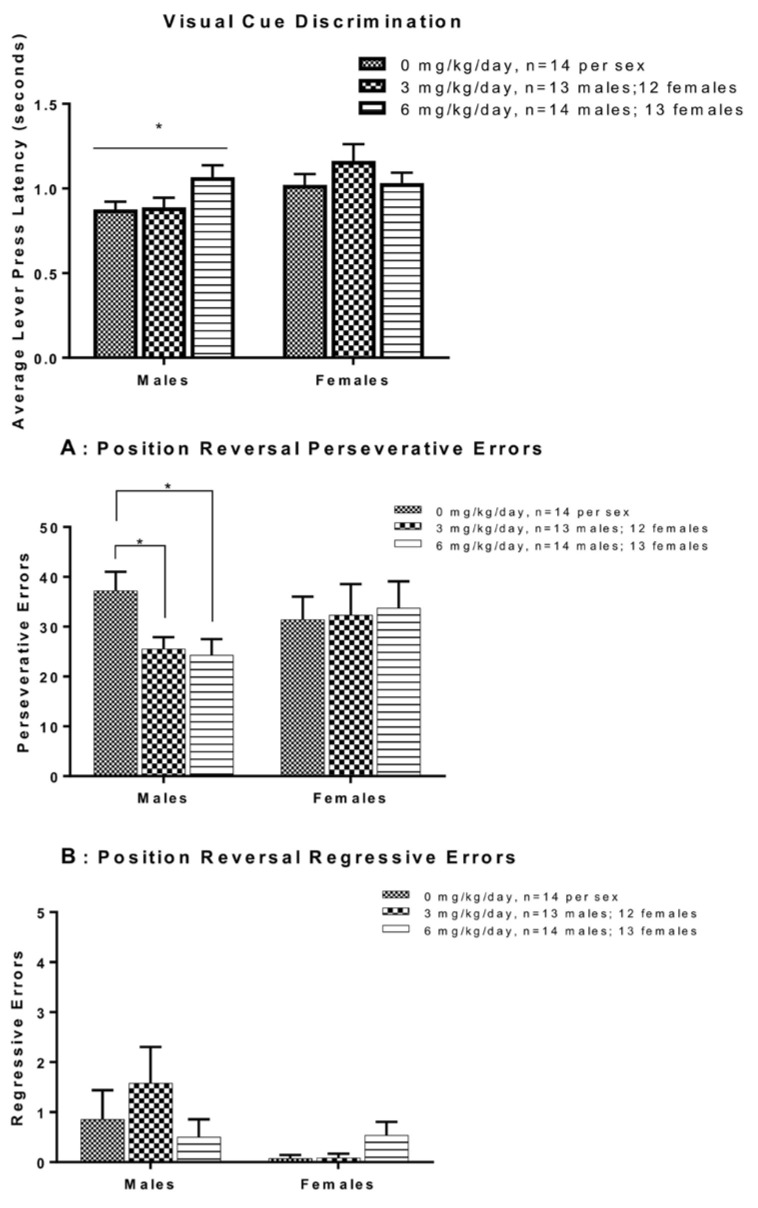
Effects of PCB exposure on cognitive flexibility and decision-making in male and female Long-Evans rats. Results are reported as mean ± SEM for all measures. “*” denotes a significant p value of *p* < 0.05. (**Top**) The average lever press latency of males and females during the visual cue discrimination phase of the set-shifting task. (**A**,**B**) The types of errors made during the reversal phase of the set-shifting task. Reprinted from [248], with permission from Elsevier.

Male and female Long-Evans rats exposed to the Fox River Mixture [244,245,246,247] orally exhibited changes in cognitive flexibility and decision-making. In the set-shifting test during the visual cue discrimination phase, there were no significant effects of PCB exposure on total errors to criterion in either sex [248]. In contrast, a significant increase in trial omissions was observed among male rats in the 6 mg/kg group compared to both the 0 mg/kg and 3 mg/kg groups [248]. Female rats exhibited no exposure-related differences in omissions or errors. A significant main effect of exposure on lever press latency was also observed in male rats during this phase, with the 6 mg/kg group taking longer to respond (Figure 14, Top) [248].

In the position discrimination (set-shift) phase, there were no significant effects of exposure on total errors to criterion in Long-Evans rats exposed to the Fox River Mixture, irrespective of sex [248]. Similarly, no group differences were observed during the first 20 trials, during which reinforcement followed the previous visual cue rule. Error subtype analysis (perseverative, regressive, and never-reinforced errors) during the set-shift phase also revealed no significant differences across exposure groups for either sex [248].

In the position reversal phase, male rats exposed to the Fox River Mixture made significantly fewer total errors to reach criterion compared to controls [248]. No exposure-related effects were observed in female rats. Error subtype analysis during reversal learning showed that males in PCB exposure groups made significantly fewer perseverative errors than controls, with no differences in regressive errors [248] (Figure 14A,B). No differences in perseverative or regressive errors were observed in females [248].

#### 3.4.9. Impulsivity and Timing

Impulse control and response inhibition are critical aspects of executive function, often evaluated in animal models using operant-based schedules such as differential reinforcement of low rates of responding (DRL) [249]. These tests require subjects to withhold responses for a specified interval to receive reinforcement, providing a measure of impulsivity and behavioral regulation [249]. Performance on DRL schedules can reflect functional integrity of prefrontal cortical and serotonergic systems [250]. Schedule-specific metrics such as total lever presses, efficiency ratios, and performance across increasing delay intervals (1 to 15 s) are used to detect subtle impairments in timing, motor control, and inhibitory capacity [250]. DRL tasks are valuable tools for assessing the long-term effects of exposures to environmental pollutants, such as PCBs, on cognitive and executive functions.

Male and female Long-Evans rats exposed to the Fox River Mixture orally were evaluated for DRL to assess impulse control. During initial DRL training at the 1 s interval (DRL1), there were no significant effects of PCB exposure on total lever presses or response efficiency in either sex [248]. In the 5 s interval phase (DRL5), both males and females improved performance across sessions; however, there were no exposure-related differences [248].

In the 10 s interval phase (DRL10), there was no main effect of session or exposure in male and female rats. A significant session × exposure interaction was observed for total lever presses in males, with a steeper decline in responding across sessions in the 3 mg/kg group compared to other groups; however, no differences were observed in the 0 or 6 mg/kg groups [248]. Females exhibited a significant session × exposure interaction, but there were no differences within groups across sessions. Efficiency ratios during DRL10 did not differ by exposure in either sex [248].

During the 30-session 15 s interval phase (DRL15), no significant effects of PCB exposure were detected in total presses or efficiency ratios across five-session blocks in either sex. In the DRL extinction phase, where reinforcement was withheld, no significant exposure effects were observed in either sex [248].

## 4. Conclusions

### 4.1. Neurotoxic Outcomes

**Figure 15:**
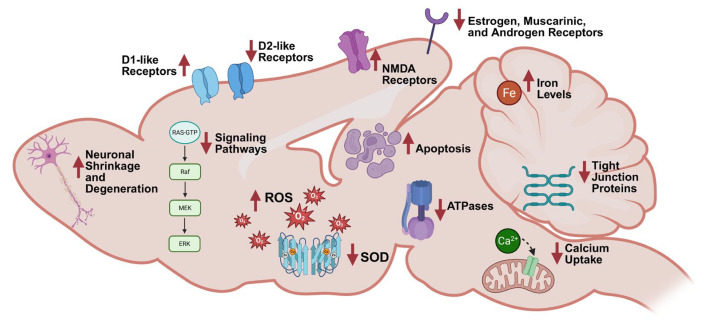
Summary of effects of PCB exposure on biochemical outcomes in the rodent brain. Red arrows indicate changes in direction. ROS: Reactive Oxygen Species. SOD: Superoxide Dismutase. NMDA: N-methyl-D-aspartate. Locations of images do not necessarily represent spatial location. Created with BioRender.com.

Collectively, the studies reviewed provide robust laboratory evidence that polychlorinated biphenyl (PCB) exposure causes widespread neurotoxic effects in rodent models, affecting histological, molecular, and biochemical endpoints across multiple brain regions (Figure 15). Structural damage, including neuronal degeneration and regional atrophy, is consistently reported in male animals, while data in females remain limited. Disruption of calcium signaling, induction of oxidative stress, and activation of apoptotic pathways were common findings, alongside marked alterations in neurotransmitter levels, receptor expression, and mRNA expression. Notably, PCBs impair dopaminergic, serotonergic, and glutamatergic systems and interfere with intracellular signaling cascades essential for synaptic function and neuronal survival. Changes in iron homeostasis, amino acid balance, and neuroinflammatory mediators further support the complex nature of PCB-induced neurotoxicity. Differences in outcomes are shaped by several key experimental factors. The PCB congener or mixture studied influences the type and severity of effects. Dose also plays a critical role, as higher exposure levels tend to yield more drastic effects, while lower doses often yield subtle or no effects. The exposure route also contributes to variability, with inhalation, oral, and i.p. exposures resulting in sometimes contradictory effects. Finally, the timing of exposure can determine whether effects manifest acutely or persist chronically. Acute exposures can transiently increase molecular responses in the brain, while prolonged exposures lead to lasting effects, such as hyperactivity in the weeks following PCB exposure [251]. Importantly, many outcomes demonstrate brain region–specific and sex-dependent variability, underscoring the need to consider sex as a biological variable in PCB neurotoxicity studies. These findings highlight the complex mechanisms by which PCBs disrupt nervous system function, with implications for understanding the risk of neurodevelopmental and neurodegenerative disorders following PCB exposure.

### 4.2. Behavioral Outcomes

**Figure 16:**
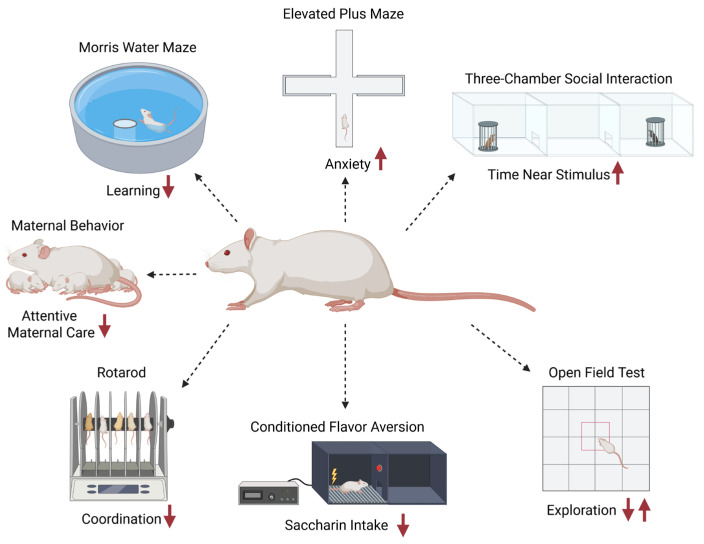
Summary of effects of PCB exposure on behavioral outcomes in rodents. Red arrows indicate changes in direction. Created with BioRender.com. See Table 7 for a summary of behavioral assays, associated domains, implicated brain regions, and proposed molecular mechanisms underlying PCB-induced neurobehavioral alterations in rodent models.

Additionally, PCBs can significantly disrupt behavioral functions in adolescent or adult rodents, with effects varying by congener or mixture, dose, exposure route, sex, and timing. PCB exposure often led to changes across various outcomes, including locomotion, anxiety-like behavior, learning and memory, motor coordination, social interaction, operant conditioning, maternal behavior, cognitive flexibility, and impulsivity, indicating dysfunction in the central nervous system following PCB exposure (Figure 16). However, the consistency of these outcomes across studies is mixed. For example, some investigations report increased locomotion following exposure, while others observe reduced or unchanged activity, even under comparable exposure conditions, suggesting that methodological variables (e.g., test apparatus, lighting, starting position) strongly influence outcomes. Motor coordination deficits have been observed in some rotarod studies, although these findings are not consistently replicated. Anxiety-related behaviors and patterns of social interaction also vary across studies, with some showing increased avoidance or changed preferences, while others indicate no impact of PCB exposure. Notably, behavioral effects were often sex-specific and exposure-dependent, with several studies reporting differing outcomes based on dose or the specific testing phase. Some effects, such as increased activity or changes in social preferences, indicate hyperactivity or disinhibition, whereas others demonstrate memory impairments following PCB exposure. Despite variability across studies, this review demonstrates that PCB exposure of adolescent or adult rodents adversely affects neurobehavioral health.

### 4.3. Limitations

Despite evidence of PCB-induced neurotoxicity in adolescent or adult rodents, several critical gaps remain that limit mechanistic understanding and the translation of findings to human health and risk assessment. A major limitation is the small number of studies using female animals, despite evidence of sex-specific variability in outcomes [252]. This imbalance of studies using male rodents compared to those including females likely reflects a historical bias toward the use of male animals in toxicology studies to avoid confounding by estrous cycle-related variability. Consequently, the scarcity of data on females limits our understanding of sex-specific susceptibility and may obscure distinct neurotoxic mechanisms. Furthermore, there is often a disconnect between biochemical endpoints and behavioral assessments, with few studies integrating these measures in the same study to directly link mechanistic alterations to functional deficits (Table 7). Moreover, behavioral assessments themselves are frequently limited to a narrow range of tests, often focusing on gross locomotor or anxiety-like measures without incorporating sensitive, domain-specific paradigms for learning, memory, sensory processing, or executive function [253]. The lack of standardized testing and laboratory variability further limits comparison across studies [253]. Application of advanced techniques, such as spatial transcriptomics, proteomics, and metabolomics, would enable high-resolution characterization of PCB-induced molecular and cellular changes across brain regions. Dose–response relationships also remain incompletely defined, with limited integration of congener or mixture composition, dose, exposure timing, and route of administration into study designs, particularly for environmentally relevant, chronic low-dose exposures. While oxidative stress, calcium signaling, neurotransmitter systems, and apoptotic pathways are frequently implicated, causal links between these mechanisms and behavioral outcomes remain unclear. This highlights the need for multi-omics integration to identify key drivers of toxicity [254,255]. In addition, alternatives to traditional animal models, such as human-derived brain organoids and microphysiological systems, offer promising paths to reduce animal use and generate human-specific mechanistic insights [256,257]. Finally, there is a lack of longitudinal studies tracking neurobiological and behavioral outcomes across the lifespan, which are essential to assess the persistence or progression of PCB-induced effects, including potential contributions to aging-related neurodegeneration.

**Table 7:** Summary of behavioral assays, associated domains, implicated brain regions, and proposed molecular mechanisms underlying PCB-induced neurobehavioral alterations in rodent models. This table integrates findings across major behavioral paradigms, including locomotor, anxiety-like, cognitive, motor, social, and maternal behaviors, and links observed outcomes to key neuroanatomical targets and molecular pathways. Proposed mechanistic interpretations provide cross-scale connections between cellular/molecular disruptions and system-level behavioral manifestations.

Behavioral Test	Behavioral Domain	Key Brain Regions Implicated	Principal Molecular Pathways/ Mechanisms	Proposed Mechanistic Interpretation (Cross-Scale Link)	References
OFT	Locomotor and Exploration; Anxiety-Like Behavior	Striatum, Prefrontal Cortex, Amygdala	Dopaminergic signaling disruption; serotonergic/GABAergic imbalance; Ca^2+^ dysregulation	Altered dopaminergic tone and excitatory–inhibitory imbalance led to hyperactivity or increased thigmotaxis indicative of anxiety-like responses.	[258,259,260]
EPM	Anxiety-Like Behavior	Amygdala, Hippocampus	Serotonergic and GABAergic disruption; oxidative stress	Neurochemical imbalance enhances amygdala excitability, increasing anxiety and reducing open-arm exploration.	[187,261,262]
Light–Dark Box	Anxiety-Like Behavior	Amygdala, Prefrontal Cortex	Serotonergic and GABAergic imbalance; neuroinflammation	Heightened inflammatory and neurotransmitter dysregulation promotes avoidance of the light zone and risk-averse exploration.	[263,264,265]
MWM	Learning and Memory	Hippocampus, Cortex	Oxidative stress; Ca^2+^ homeostasis disruption; BDNF–TrkB axis impairment; thyroid hormone signaling	Oxidative injury and reduced neurotrophic support impair hippocampal plasticity and spatial learning.	[266,267,268]
8-Arm Radial Maze	Learning and Memory	Hippocampus, Prefrontal Cortex	Oxidative stress; mitochondrial dysfunction	Energy deficits and oxidative damage reduce accuracy and increase latency in memory retrieval.	[198,269,270]
CFA	Learning and Memory	Insular Cortex, Amygdala	Synaptic plasticity gene dysregulation; oxidative stress	Impaired synaptic encoding of aversive cues due to oxidative stress and reduced immediate-early gene activation.	[271,272,273]
Rotarod	Motor Coordination and Balance	Cerebellum, Motor Cortex, Striatum	Dopaminergic disruption; mitochondrial dysfunction; apoptosis	Motor impairments arise from cerebellar mitochondrial toxicity and dopaminergic neuron loss.	[274,275,276]
Three-Chamber Social Interaction	Social and Sociosexual Behavior	Prefrontal Cortex, Hippocampus, Amygdala	BDNF–TrkB axis disruption; neuroinflammation	Reduced neurotrophic signaling and cytokine-mediated synaptic pruning impair social approach behavior.	[217,277,278]
Social Novelty Test	Social and Sociosexual Behavior	Hippocampus, Prefrontal Cortex	BDNF–TrkB impairment; oxidative stress	Diminished hippocampal plasticity and oxidative injury impair discrimination between familiar and novel animals.	[279,280,281]
USV	Social and Sociosexual Behavior	Amygdala, Periaqueductal Gray	Synaptic plasticity gene dysregulation; neuroinflammation	Altered synaptic signaling and neuroimmune activation disrupt communication and social bonding cues.	[218,282,283]
Operant Conditioning	Operant Conditioning and Behavioral Flexibility	Striatum, Prefrontal Cortex	Dopaminergic disruption; oxidative stress	Impaired reward processing and task acquisition due to frontostriatal dysfunction.	[284,285,286]
Maternal Care	Reproductive and Maternal Behavior	Hypothalamus, Prefrontal Cortex	Thyroid hormone disruption; dopaminergic signaling	Endocrine disruption and altered reward circuits reduce pup-directed behaviors.	[232,287,288]
Set-Shifting/Reversal Learning	Cognitive Flexibility and Decision-Making	Prefrontal Cortex	Neuroinflammation; synaptic gene dysregulation	Prefrontal cytokine activation and reduced synaptic remodeling impair task- switching and adaptability.	[289,290,291]

In addition to these research gaps, several methodological considerations related to the use of large language models in the review process should be acknowledged. ChatGPT was used to assist with text summarization and data organization. Because the “improve the model for everyone” feature was not enabled, study data or text was not shared for model training, safeguarding data privacy. We acknowledge that large language models may exhibit biases reflective of their training data and have limited context retention when processing complex or lengthy text. To mitigate these limitations, all AI-generated outputs were reviewed and verified by human researchers for accuracy and consistency with the source material. Moreover, as large language model outputs are not fully reproducible due to model updates and stochastic variation, all ChatGPT responses used in data extraction were documented to promote transparency and reproducibility.

### 4.4. Future Directions and Translational Outlook

Future research should leverage emerging technologies, including spatial transcriptomics, proteomics, and metabolomics, to achieve high-resolution mapping of PCB-induced molecular and cellular alterations across brain regions and developmental stages. Integrating congener composition, dose, exposure timing, and route of administration into study designs will enhance environmental relevance and mechanistic interpretation. Key translational questions regarding PCB neurotoxicity remain: What molecular pathways at the cellular level causally link PCB exposure to behavioral outcomes, and do alterations in oxidative stress, calcium homeostasis, and neurotransmission jointly drive these effects? How do the neurotoxic mechanisms and dose–response relationships differ between legacy PCBs and inadvertently produced PCB congeners? How does the route of exposure, such as inhalation, oral ingestion, or dermal absorption, influence the neurotoxic outcomes and behavioral phenotypes observed in experimental models? Do region- and cell type–specific transcriptional responses predict distinct neurobehavioral phenotypes? Can human-derived brain organoids and microphysiological systems replicate mixture- and dose–response effects observed in vivo, thereby improving human relevance while reducing animal use? Finally, do PCB-induced neurobiological alterations persist across the lifespan or accelerate aging-related neurodegeneration? Addressing these questions through multi-omics integration and longitudinal study designs will establish a translational framework connecting mechanistic discoveries in experimental models with human health risk assessment.

## Figures and Tables

**Table 1:** Questions asked ChatGPT to feed the model with information about the topic.

ID	Question
1	What are polychlorinated biphenyls?
2	How are people exposed to PCBs?
3	How do PCBs impact health?
4	How are PCBs neurotoxic?
5	How do PCBs impact behavior?
6	How do PCBs impact neurobehavior?
7	How are PCBs studied in mice and rats?

**Table 2:** ChatGPT’s response to the request for classification of titles/abstracts.

Author/Title/Year	Mouse/Rat/Human/In Vitro Related	PCB or Related Chemicals	Neurotoxic Outcome Listed	Not Related	Explanation
Nilsson, C. B. et al. (2000). “2,3,7,8-tetrachlorodibenzo-p-dioxin increases serum and kidney retinoic acid levels and kidney retinol esterification in the rat.”	X	X			Involves rats and TCDD, a related chemical to PCBs.
Nilsson, E. et al. (2012). “Environmentally induced epigenetic transgenerational inheritance of ovarian disease.”	X	X			Involves rats and various environmental chemicals, including dioxins.
Ning, S. and X. Xiaobai (1997). “Reductive metabolism of 4-nitrobiphenyl by rat liver fraction.”	X				Involves rats but not related to PCBs or neurotoxic outcomes.
Ninou, I. et al. (2018). “Pharmacologic targeting of the ATX/LPA axis attenuates bleomycin-induced pulmonary fibrosis.”				X	Not related to PCBs or neurotoxic outcomes.
Nishida, N. et al. (1997). “Effects of acute and repeated exposures to Aroclor 1254 in adult rats: motor activity and flavor aversion conditioning.”	X	X	X		Involves rats, Aroclor 1254 (a type of PCB), and studies neurotoxic outcomes.

**Table 3:** Questions asked ChatGPT to extract information from each article.

ID	Question
1. PCBs	
1-1	Does the paper describe the use of PCBs? Must only be PCB congeners, not related substances. If the answer is no, do not proceed any further with the assessment.
2. Mouse/Rat	
2-1	Does the paper describe the use of mice or rats in the study?
2-2	Are mice or rats directly exposed to PCBs?
2-3	Are cell cultures from mice or rats exposed to PCBs?
2-4	Are tissues collected from mice or rats exposed to PCBs?
3. Exposure Timeframe	
3-1	Does the article describe dosing PCBs during gestation?
3-2	Does the article describe dosing PCBs during lactation?
3-3	Does the article describe dosing PCBs to the mouse/rat before postnatal day 21?
3-4	Does the article mention dosing after weaning/post-natal day 21?
3-5	If applicable, were maternal/dam tissues collected or maternal analyses done?
4. Neuro-Outcomes	
4-1	Does the paper describe analyses related to neurobehavior or neurotoxicity?
4-2	Does the paper describe behavioral analysis?
4-3	Does the paper describe gene analysis?
4-4	Does the paper describe receptor changes?

**Table 4:** ChatGPT’s response to the request for information extraction from the methods section.

Author/Title/Year	Sub-Part	Yes/No	Explanation
Coburn, C. G. et al., 2015. Permanently compromised NADPH-diaphorase activity within the osmotically activated supraoptic nucleus after in utero but not adult exposure to Aroclor 1254. Neurotoxicology, 47, 37–46.	1-1	Yes	The paper describes the use of Aroclor 1254, a commercial PCB mixture.
	2-1	Yes	The study uses male Sprague-Dawley rats.
	2-2	Yes	Rats were exposed to Aroclor 1254 through cheese puff snacks infused with the PCB mixture.
	2-3	No	The study does not describe the use of cell cultures from mice or rats exposed to PCBs.
	2-4	Yes	Brain tissues were collected from rats exposed to Aroclor 1254 for histochemical analysis.
	3-1	Yes	In utero exposure was accomplished by feeding pregnant dams cheese puff snacks infused with Aroclor 1254 for 10 days during gestation (GD 10–19).
	3-2	No	The article does not describe dosing PCBs during lactation.
	3-3	Yes	Pups were exposed to PCBs in utero and allowed to suckle until weaning at PND 22.
	3-4	Yes	Adult rats were exposed to PCBs at 3–5 months old, and some were allowed to age until 14–16 months before sacrifice.
	3-5	No	The study does not mention collecting maternal/dam tissues or conducting maternal analyses.
	4-1	Yes	The paper describes the assessment of NADPH-diaphorase activity, which is related to neurotoxicity.
	4-2	No	The paper does not describe behavioral analysis.
	4-3	No	The paper does not describe gene analysis.
	4-4	No	The paper does not describe receptor changes.

**Table 5:** Cohen’s kappa values and interpretations for inter-rater reliability of title/abstract screening selections. A: The total number of instances that both raters said were correct. B: The total number of instances that Rater 2 said was incorrect, but Rater 1 said were correct. C: The total number of instances that Rater 1 said was incorrect, but Rater 2 said were correct. D: The total number of instances that both raters said were incorrect.

Comparison	A	B	C	D	Agreement	Kappa	SE	95% CI	Interpretation
Reviewer 1 & Reviewer 2	372	71	110	2711	94.45%	0.772	0.02	0.740–0.804	Substantial Agreement
Reviewer 1 & ChatGPT	301	142	47	2774	94.21%	0.729	0.02	0.692–0.765	Substantial Agreement
Reviewer 2 & ChatGPT	297	51	185	2731	92.77%	0.675	0.02	0.637–0.714	Substantial Agreement

**Table 6:** Cohen’s kappa values and interpretations for inter-rater reliability for method screening selections. A: The total number of instances that both raters said were correct. B: The total number of instances that Rater 2 said was incorrect, but Rater 1 said were correct. C: The total number of instances that Rater 1 said was incorrect, but Rater 2 said were correct. D: The total number of instances that both raters said were incorrect.

Comparison	A	B	C	D	Agreement	Kappa	SE	95% CI	Interpretation
Reviewer 1 & Reviewer 2	30	13	15	302	92.22%	0.638	0.06	0.514–0.761	Moderate- Substantial Agreement
Reviewer 1 & ChatGPT	37	6	15	302	94.17%	0.746	0.05	0.642–0.849	Moderate- Almost Perfect Agreement
Reviewer 2 & ChatGPT	29	23	16	292	89.17%	0.536	0.07	0.407–0.664	Fair-Moderate Agreement

## Data Availability

The original data presented in the study are openly available in Iowa Research Online at 10.25820/data.008102.

## Supplementary Materials

The following supporting information can be downloaded at https://www.mdpi.com/article/10.3390/ijms262210829/s1.

## Abbreviations

The following abbreviations are used in this manuscript:

5-HIAA	5-hydroxyindoleacetic acid
AChE	Acetylcholinesterase
AhR	Aryl hydrocarbon receptor
ANOVA	Analysis of variance
Ars	Androgen receptors
AF-6	Afadin
Avpr1a	Vasopressin receptor 1a
Bad	BCL2-associated death promoter
Bax	BCL2-associated X protein
Bcl2	B-cell leukemia/lymphoma 2
BDNF	Brain-derived neurotrophic factor
Bid	BH3 interacting-domain death agonist
BNST	Bed nucleus of the stria terminalis
CAR	Constitutive androstane receptor
CAT	Catalase
CASP	Caspase
CBL	Cerebellar
CFA	Conditioned flavor aversion
cGMP	Cyclic guanosine monophosphate
Cldn5	Claudin-5
CNS	Central nervous system
COMT	Catechol-O-methyltransferase
CREB	Cyclic AMP-response element binding protein
CRF	Continuous reinforcement
Cu/Zn SOD	Cu/Zn superoxide dismutase
CYPs	Cytochrome P450 monooxygenases
DA	Dopamine
DAT	Dopamine transporter
DOPAC	3,4-dihydroxyphenylacetic acid
DRL	Differential reinforcement of low rates of responding
DRL1	DRL training at the 1 s interval
DRL10	DRL training at the 10 s interval
DRL15	DRL training at the 15 s interval
DRL5	DRL training at the 5 s interval
EAC1	Excitatory amino acid carrier 1
EPM	Elevated Plus Maze
ERK	Extracellular signal-regulated kinase
ERs	Estrogen receptors
EXT	Extinction
Fas	Tumor necrosis factor receptor superfamily member 6
FasL	Fas ligand
FI	Fixed interval
GLAST	Glutamate aspartate transporter
GLT-1	Glutamate transporter 1
GPV	Glial plasmalemmal vesicle
GPx	Glutathione peroxidase
GR	Glutathione reductase
GSTs	Glutathione-S-transferases
HO-1	Heme oxygenase-1
HVA	Homovanillic acid
i.p.	Intraperitoneal
Jam3	Junction adhesion molecule-3
LTME	Long-term memory error
MAP	Microtubule-associated protein
MeA	Medial amygdala
Mn SOD	Manganese SOD
MR	Muscarinic receptor
MWM	Morris water maze
NAPDH-d	NADPH-diaphorase
NE	Norepinephrine
Nefl	Neurofilament light chain
NF-kB	Nuclear factor kappa B
NMDA	N-methyl-D-aspartate
NOS	Nitric oxide synthase
NTKRB	Neurotrophin tyrosine kinase receptor B
Ocln	Occludin
OFT	Open Field Test
OH-PCBs	Hydroxylated metabolites
Oprm1	mu opioid receptor
PCBs	Polychlorinated biphenyls
PKA	Protein Kinase A
PKC	Protein Kinase C
PPARs	Peroxisome proliferator-activated receptors
PRISMA	Preferred Reporting Items for Systematic Reviews and Meta-Analysis
PVN	Paraventricular nucleus
PXR	Pregnane X receptor
RNS	Reactive nitrogen species
ROS	Reactive oxygen species
RyRs	Ryanodine receptors
SAM	School Air Mixture
SNpc	Substantia nigra pars compacta
SON	Supraoptic nucleus
STME	Short-term memory errors
STR	Striatal
SULTS	Sulfotransferases
TBARS	Thiobarbituric acid reactive substances
TH	Tyrosine hydroxylase
TH−	TH-negative
TH+	Tyrosine hydroxylase-positive
TME	Total memory error
TNFa	Tumor necrosis factor alpha
UGTs	UDP-glucuronosyltransferases
U.S.	United States
USVs	Ultrasonic vocalizations
VMH	Ventromedial hypothalamus
VTA	Ventral tegmental area
ZO	Zona Occludens

## Appendix A

**Table A1:** Summary of Studies Investigating Neurotoxic Outcomes in Sprague Dawley Rats Following PCB Exposure.

PCB Source	Dose (mg/kg/day) ^a^	Exposure Route	Vehicle	Exposure Period	Age	N ^b^	Sex	Brain Region	Analysis	Result ^c^	Ref
A1254 ^d^	62.1 ± 17.7 μg/m^3^	Inh ^e^	LA ^f^	28 days	Adult (239 ± 8g)	8	F ^g^	Whole Brain	Histology H&E ^h^	- Gross histopathological changes in HC ^i^ - Features of necrosis or apoptosis	[24]
A1254 A1221	45.5 ± 5.9 μg/m^3^	Inh	LA	6 d/w ^j^ for 13 weeks	Adult (241 ± 9g)	S ^k^: 8 E ^l^: 12	F	Whole Brain	Histology H&E	- Histopathological changes	[25]
A1254	62.1 ± 17.7 μg/m^3^	Inh	LA	28 days	Adult (239 ± 8g)	8	F	Whole Brain	OS ^m^ Markers	↓ ROS ^n^/RNS ^o^	[24]
A1254 A1221	45.5 ± 5.9 μg/m^3^	Inh	LA	6 d/w for 13 weeks	Adult (241 ± 9g)	S: 8 E: 12	F	Whole Brain	OS Markers	- ROS/RNS	[25]
A1221	0 or 1	IP ^p^	4% DMSO ^q^	PND24 ^r^, 26, 28	PND24	9–12	M ^s^/F	BNST ^t^ MeA ^u^ PVN ^v^ VMH ^w^	DNA Methylation	- DNA methylation	[163]
A1254	0 or 25	PO ^x^	CO ^y^ on VW ^z^	3 days, or 1, 2, or 8 weeks	12 weeks	3–4	M	Striatal Dialysate	DA ^aa^ Concentrations	↑ DA after 3 days ↓ DA after 1, 2, and 8 weeks	[132]
A1254	0 or 25	PO	CO on VW	3 days, or 1, 2, or 8 weeks	12 weeks	3–6	M	ST ^bb^	DA Concentrations	- DA levels	[132]
A1254 A1221	45.5 ± 5.9 μg/m^3^	Inh	LA	6 d/w for 13 weeks	Adult (241 ± 9g)	S: 8 E: 12	F	ST	DA and DOPAC ^cc^ Concentrations	- DA and DOPAC levels	[25]
PCB 180	0, 19.4, 64.3, 194, 643, 2000, 6430 or 13000	PO	CO	28 days	6 weeks	5	M/F	CE ^dd^	DA and Nicotinic Receptors	- Specific binding to DRD1 ^ee^/DRD5 receptors - High or low affinity sites on nicotinic receptor	[119]
PCB 180	0, 19.4, 64.3, 194, 643, 2000, 6430 or 13,000	PO	CO	28 days	6 weeks	5	M/F	CE	Brain AA ^ff^	↓ GSH ^gg^ in males - γ-Amino butyric acid, aspartate, glutamate, serine, glycine, taurine, glutamine, alanine,	[119]
A1254 A1221	45.5 ± 5.9 μg/m^3^	Inh	LA	6 d/w for 13 weeks	Adult (241 ± 9g)	S: 8 E: 12	F	Whole Brain	Cytokine and Chemokine	- Cytokine or chemokine concentrations	[25]
A1221	0 or 1	IP	4% DMSO	PND24, 26, 28	P24	9–12	M/F	PoA ^hh^	Gene Expression	↓ Expression of *Ar* ^ii^ and *Oprm**1* ^jj^ in males	[163]
A1221	0 or 1	IP	4% DMSO	PND24, 26, 28	P24	9–12	M/F	PFC ^kk^	Gene Expression	↑ Expression of *Oprm1* in males	[163]
A1221	0 or 1	IP	4% DMSO	PND24, 26, 28	P24	9–12	M/F	Lateral septum	Gene Expression	↓ Expression of *Avpr1a* ^ll^ in males	[163]
A1221	0 or 1	IP	4% DMSO	P24, 26, 28	P24	9–12	M/F	PFC	Gene × behavior interactions	↑ Correlation of no-hormone stimulus time and *Oprm1*	[163]
A1221	0 or 1	IP	4% DMSO	P24, 26, 28	P24	9–12	M/F	BNST MeA PVN VMH	Gene Expression	- Gene expression	[163]
A1254 A1221	45.5 ± 5.9 μg/m^3^	Inh	LA	6 d/w for 13 weeks	Adult (241 ± 9g)	S: 8 E: 12	F	Brain	RNAseq and Pathway Analysis	↑ 274 genes ↓ 58 genes Pathways altered NS, CD, VF, + IR	[25]
A1254	0 or 30	PO	CO in Cheeto	14 days	3–5 months	3–6	M	SON ^mm^	Hyperosmotic Challenge and NOS Activity	- NAPDH-d density	[151]
PCB 153	0 or 20	PO	CO	GD10-GD16 ^nn^	15 weeks	5	F	CC CE HC ST	Muscarinic Receptor	↑ MR ^oo^ density in CC ↓ MR density in CE - MR density in HC or ST	[164]

^a^ Unless otherwise noted. ^b^ N, Number of animals per group and sex. ^c^ (-) no change; (↑) significant increase; (↓) significant decrease. ^d^ A, Aroclor. ^e^ Inh, Inhalation. ^f^ LA, Lab air. ^g^ F, Female. ^h^ H&E, Hematoxylin and eosin staining. ^i^ HC, Hippocampus. ^j^ d/w, Days per week. ^k^ S, Sham. ^l^ E, Exposed. ^m^ OS, Oxidative stress. ^n^ ROS, Reactive oxygen species. ^o^ RNS, Reactive nitrogen species. ^p^ IP, Intraperitoneal injection. ^q^ DMSO, Dimethyl sulfoxide. ^r^ PND, Postnatal day. ^s^ M, Male. ^t^ BNST, Bed nucleus of the stria terminalis. ^u^ MeA, Medial amygdala. ^v^ PVN, Paraventricular nucleus. ^w^ VMH, Ventromedial hypothalamus. ^x^ PO, Oral. ^y^ CO, Corn oil. ^z^ VW, Vanilla wafer. ^aa^ DA, Dopamine. ^bb^ ST, Striatum. ^cc^ DOPAC, 3,4-dihydroxyphenylacetic acid. ^dd^ CE, Cerebellum. ^ee^ DRD, Dopamine receptor D. ^ff^ AA, Amnio acid. ^gg^ GSH, Glutathione. ^hh^ PoA, Preoptic area. ^ii^ *Ar*, Androgen receptor. ^jj^ *Oprm1*, Mu opioid receptor. ^kk^ PFC, Prefrontal cortex. ^ll^ *Avpr1a*, Arginine vasopressin receptor 1A. ^mm^ SON, Supraoptic nucleus. ^nn^ GD, Gestational day. ^oo^ MR, Muscarinic receptor.

**Table A2:** Summary of Studies Investigating Neurotoxic Outcomes in Wistar Rats Following PCB Exposure.

PCB Source	Dose (mg/kg/day)	Exposure Route	Vehicle	Exposure Period	Age	N ^a^	Sex	Brain Region	Analysis	Result ^b^	Ref
A1254 ^c^	0 or 2	IP ^d^	CO ^e^	30 days	Adult (180–200 g)	6	M ^f^	CC ^g^ CE ^h^	Histology Methylene Blue	↑ Degenerated neurons	[106]
A1254	0 or 2	IP	CO	30 days	Adult (180–200 g)	6	M	CE	Histology H&E ^i^	↑ Shrinkage and degeneration of Purkinje neurons	[105]
A1254	0 or 2	IP	CO	30 days	Adult (180–200 g)	4	M	CC	Histology H&E/Luxol Fast Blue	↑ Pyknotic CB ^j^, NC ^k^, IBA ^l^ ↓ Pyramidal, Betz, stellate cells, myelin density	[107]
A1254	0 or 2	IP	CO	30 days	PND90 ^m^	6	M	CC	Histology H&E	↑ Neuronal shrinkage and degeneration ↑ Pyknotic nuclei with PPNS ^n^	[104]
A1254	0 or 2	IP	CO	30 days	PND90	6	M	HC ^o^	Histology Cresyl Fast Violet	↑ Disruption in CA4 ^p^	[110]
A1254	0 or 2	IP	CO	30 days	PND90	6	M	HC	Histology H&E	↑ Degeneration of pyramidal cells ↑ Neuronal shrinkage of HC layer	[109]
A1254	0 or 2	IP	CO	30 days	PND90	6	M	HC	Histology H&E	↑ Degeneration of pyramidal cells ↑ Neuronal shrinkage of HC layer	[108]
A1254	0 or 2	IP	CO	30 days	PND90	6	M	CE CC HC	Histology H&E	↑ Pyknotic nuclei with PPNS in CC ↑ Degeneration of pyramidal cells in HC ↑ Purkinje cells in CE with shrinkage, degeneration, and moderate atrophy	[103]
A1254	0 or 10	PO ^q^	RO ^r^	14 days	Young Adult (200–250 g)	DND ^s^	M	HC	Histology Toluidine Blue	↓ Number of neurons	[111]
A1254	0 or 10	PO	RO	14 days	Young Adult (200–250 g)	DND	M	FBC ^t^ HC	Transmission Electron Microscopy	↑ Damaged neuron nuclei in FBC and HC ↑ RER ^u^ damage in HC	[111]
A1254	0 or 2	IP	CO	30 days	PND90	6	M	CC	Cacna1d ^v^ Expression	↑ mRNA expression of Cacna1d	[104]
A1254	0 or 2	IP	CO	30 days	PND90	6	M	HC	Cacna1d Expression	↓ mRNA expression of Cacna1d	[109]
A1254	0 or 2	IP	CO	30 days	Adult (180–200 g)	6	M	CC	Protein Expression	↑ NMDA ^w^ receptor expression ↓ PKA^x^α levels ↑ Calpain expression ↑ NcaCP ^y^ level	[113]
A1254	0 or 10	PO	RO	14 days	Young Adult (200–250 g)	DND	M	HC	Ca^2+^ Efflux	↓ NMDA Ca^2+^ release	[111]
A1254	0 or 2	IP	CO	30 days	Adult	6	M	CE	OS ^z^ Markers	↑ LP ^aa^, HP ^bb^, and PC ^cc^	[105]
A1254	0 or 2	IP	CO	30 days	PND90	6	M	CE HC CC	mRNA Expression	↓ Expression of Cu/Zn SOD ^dd^ ↓ Expression of GPx-4 ^ee^	[103]
A1254	0 or 2	IP	CO	30 days	PND90	6	M	CC	OS Markers	↑ LP, HP, and PC	[104]
A1254	0 or 2	IP	CO	30 days	PND90	6	M	HC	OS Markers	↑ LP, HP, HR ^ff^, and TBARS ^gg^	[110]
A1254	0 or 2	IP	CO	30 days	PND90	6	M	HC	OS Markers	↑ LP, HP, and PC	[116]
A1254	0 or 2	IP	CO	30 days	PND90	6	M	HC	OS Markers	↑ LP, HP, and PC	[108]
A1254	0 or 2	IP	CO	30 days	PND90	6	M	CE HC CC	OS Markers	↑ LP and HR ↓ Glutathione	[118]
A1254	0 or 2	IP	CO	30 days	PND90	6	M	CE HC CC	OS Markers	↑ LP, HP, and HR ↓ Glutathione	[117]
A1254	0 or 2	IP	CO	30 days	PND90	6	M	HC	Antioxidant Enzymes	↓ SOD, CAT ^hh^, GPx, GST ^ii^, and GR ^jj^	[110]
A1254	0 or 2	IP	CO	30 days	PND90	6	M	CE HC CC	Antioxidant Enzyme Activity	↓ Total SOD, Cu/Zn and Mn SOD ↓ Activity of GPx	[103]
A1254	0 or 2	IP	CO	30 days	PND90	6	M	HC	DNA Fragmentation	Streak of fragmentation	[108]
A1254	0 or 2	IP	CO	30 days	PND90	6	M	CC	ATPase Activity	↓ Na/K, Ca, and Mg ATPase activity	[104]
A1254	0 or 2	IP	CO	30 days	Adult (180–200 g)	6	M	CE	ATPase Activity	↓ Na/K, Ca, and Mg ATPase activity	[105]
A1254	0 or 2	IP	CO	30 days	PND90	6	M	HC	ATPase Activity	↓ Na/K, Ca, and Mg ATPase activity	[109]
A1254	0 or 2	IP	CO	30 days	PND90	6	M	CE CC HC	ATPase Activity	↓ Na/K, Ca, and Mg ATPase activity	[117]
A1254 A1260	0, 500 or 1000	PO	CO	1 day	Adult (250–300 g)	36	M	Caudate LOT ^kk^	Dopamine Metabolites	↓ DA and DOPAC in caudate ↓ DOPAC/DA ratio in LOT - DA, DOPAC in LOT - HVA ^ll^, HVA/DA ratio	[134]
A1254	0, 500 or 1000	PO	AOC ^mm^	30 days	Adult (350–375 g)	9	M	ST ^nn^	Dopamine Metabolites	↓ DA, HVA, and DOPAC/DA, HVA/DA ratios	[133]
A1254	0, 500 or 1000	PO	AOC	30 days	Adult (350–375 g)	9	M	LOT	Dopamine Metabolites	↓ DOPAC, HVA, and DOPAC/DA, HVA/DA ratios - DA levels	[133]
A1254 A1260	0, 500 or 1000	PO	CO	1 day	Adult (250–300 g)	36	M	FC ^oo^	Neurotransmitter Concentrations	↓ 5-HT ^pp^ ↑ 5-HIAA ^qq^, 5-HIAA/5-HT ratio	[139]
A1254 A1260	0, 500 or 1000	PO	CO	1 day	Adult (250–300 g)	36	M	HC	Neurotransmitter Concentrations	↓ 5-HT ↑ 5-HIAA/5-HT ratio levels - 5-HIAA levels	[139]
A1254 A1260	0, 500 or 1000	PO	CO	1 day	Adult (250–300 g)	36	M	LOT	Neurotransmitter Concentrations	↑ 5-HT and 5-HIAA - 5-HIAA/5HT ratio	[139]
A1254 A1260	0, 500 or 1000	PO	CO	1 day	Adult (250–300 g)	36	M	BS ^rr^ HT ^ss^	Neurotransmitter Concentrations	↑ 5-HIAA/5HT ratio - 5-HT and 5-HIAA	[139]
A1254	0, 500 or 1000	PO	AOC	30 days	Adult (350–375 g)	9	M	ST LOT	Neurotransmitter Concentration/Activity	- NE ^tt^ or 5-HT	[133]
A1254 A1260	0, 500 or 1000	PO	CO	1 day	Adult (250–300 g)	36	M	DFC ^uu^ HC	NE Concentration	↓ NE	[138]
A1254 A1260	0, 500 or 1000	PO	CO	1 day	Adult (250–300 g)	36	M	MBH ^vv^ BS	NE Concentration	- NE	[138]
A1254	0 or 2	IP	CO	30 days	Adult (180–200 g)	6	M	CE	DR ^ww^ Expression	↑ DRD1 ^xx^ and DRD5 ^yy^ mRNA and protein ↓ DRD2 ^zz^, DRD3 ^aaa^ and DRD4 ^bbb^ mRNA and protein	[105]
A1254	0 or 2	IP	CO	30 days	PND90	6	M	CC	DR Expression	↑ DRD1 and DRD5 mRNA ↓ DRD2, DRD3, and DRD4 mRNA	[104]
A1254	0 or 2	IP	CO	30 days	PND90	6	M	HC	DR Expression	↑ DRD1 and DRD5 mRNA and protein ↓ DRD2, DRD3 and DRD4 mRNA and protein	[109]
A1254	0 or 2	IP	CO	30 days	Adult (180–200 g)	6	M	CE	TH ^ccc^ Expression	↓ TH protein - TH mRNA	[105]
A1254	0 or 2	IP	CO	30 days	PND90	6	M	HC	TH expression	- TH mRNA	[109]
A1254	0 or 2	IP	CO	30 days	Adult (180–200 g)	6	M	CC CE	Apoptotic Gene Expression	↑ NF-kB ^ddd^ expression	[106]
A1254	0 or 2	IP	CO	30 days	Adult (180–200 g)	4	M	CC	Bcl2 ^eee^ Signaling Protein	↑ Bid ^fff^, Bad ^ggg^, Bax ^hhh^, CASP3 ^iii^, CASP9 ^jjj^ ↓ Bcl2	[107]
A1254	0 or 2	IP	CO	30 days	PND90	6	M	HC	Bcl2 Signaling mRNA	↑ Bid, Bad, Bax, CASP3, CASP9 ↓ Bcl2	[108]
A1254	0 or 2	IP	CO	30 days	Adult (180–200 g)	6	M	CC CE	Bcl2 Signaling mRNA	↑ Bad ↓ Bcl2	[106]
A1254	0 or 2	IP	CO	30 days	PND90	6	M	HC	Apoptotic Signaling Expression	↑ TNFa ^kkk^, NF-kB, Fas ^lll^, and FasL ^mmm^ mRNA and protein	[108]
A1254	0 or 2	IP	CO	30 days	Adult (180–200 g)	6	M	CC CE	Apoptotic Gene Expression	↑ FasL and CASP8 ^nnn^	[106]
A1254	0 or 2	IP	CO	30 days	PND90	6	M	HC	Caspase Expression and Activity	↑ CASP8, 9, and 3 mRNA and protein ↑ CASP3 activity	[108]
A1254	0 or 10	PO	RO	14 days	Young Adult (200–250 g)	DND	M	HC micro-dialysate	cGMP and Amino Acid Levels	↓ NDMA-evoked cGMP and Glu ↓ Basal Glu, Arg, and Gln - Basal cGMP, Tau, Asp	[111]
A1254	0 or 2	IP	CO	30 days	PND90	6	M	CC	Creatine Kinase Activity	↓ Creatine kinase activity	[104]
A1254	0 or 2	IP	CO	30 days	PND90	6	M	CE CC HC	Creatine Kinase Activity	↓ Creatine kinase activity	[118]
A1254	0 or 2	IP	CO	30 days	Adult (180–200 g)	6	M	CE	Functional Enzyme Activity	↓ Creatine kinase A and AChE ^ooo^	[105]
A1254	0 or 2	IP	CO	30 days	PND90	6	M	CC	AChE Activity	↓ Mean AChE activity	[104]
A1254	0 or 2	IP	CO	30 days	PND90	6	M	CE CC HC	AChE Activity	↓ Specific activity of AChE	[117]
A1254	0 or 2	IP	CO	30 days	Adult (180–200 g)	6	M	CC	Neurotrophin Signaling Expression	↓ Expression of GLAST ^ppp^, BDNF ^qqq^, NTRKB ^rrr^, MAP1 ^sss^, ERK1 ^ttt^, ERK2 ^uuu^, CREB ^vvv^	[113]
A1254	0 or 10	PO	RO	14 days	Young Adult (200–250 g)	DND	M	FB CE	Glutamate Transporter Expression	↓ GLT-1 ^www^ mRNA and protein in FB - GLT-1 in CE - GLAST in CE and FB	[141]
A1254	0 or 10	PO	RO	14 days	Young Adult (200–250 g)	DND	M	Whole Brain SS ^xxx^ and GS ^yyy^	[^3^H] Glutamate Transport	↑ Glutamate uptake in SS ↓ Glutamate uptake in GS ↓ Accumulation of amino acids ↑ KCl-stimulated release of glutamate - Rate of [^3^H] glutamate release	[141]
A1254	0 or 10	PO	RO	14 days	Young Adult (200–250 g)	DND	M	FB CE	Glutamate Transporter Expression	↑ EAAC1 ^zzz^ protein in FB ↓ EAAC1 mRNA in FB ↓ EAAC1 mRNA and protein in CE	[141]
A1254	0 or 2	IP	CO	30 days	PND90	6	M	HC	mRNA Expression	↓ Estrogen α and β ↓ Occludin, claudin, ZO1 ^aaaa^ and 2, AF6 ^bbbb^ and JAM ^cccc^	[116]
A1254	0 or 2	IP	CO	30 days	PND90	6	M	HC	Protein Expression	↓ Ras ^dddd^ + Raf ^eeee^ protein levels	[116]
A1254	0 or 2	IP	CO	30 days	Adult (180–200 g)	6	M	CC CE	mRNA and Protein Expression	↓ Nefl ^ffff^ mRNA and protein	[106]

^a^ N, Number of animals per group and sex. ^b^ (-) no change; (↑) significant increase; (↓) significant decrease. ^c^ A, Aroclor. ^d^ IP, Intraperitoneal injection. ^e^ CO, Corn oil. ^f^ M, Male. ^g^ CC, Cerebral cortex. ^h^ CE, Cerebellum. ^i^ H&E, Hematoxylin and eosin staining. ^j^ CB, Cell body. ^k^ NC, Nonhomologous cytoplasm. ^l^ IBA, Intense basophilic appearance. ^m^ PND, Postnatal day. ^n^ PPNS, Predominant peri-neuronal spaces. ^o^ HC, Hippocampus. ^p^ CA4, Cornu Ammonis 4. ^q^ PO, Oral. ^r^ RO, Rape oil. ^s^ DND, Did not describe. ^t^ FBC, Forebrain cortex. ^u^ RER, Rough endoplasmic reticulum. ^v^ Cacna1d, Calcium channel, voltage dependent L-type, α 1 D subunit. ^w^ NMDA, N-methyl-D-aspartate. ^x^ PKA, Protein kinase A. ^y^ NCaCP, N-type Ca^2+^ channel protein α1B. ^z^ OS, Oxidative Stress. ^aa^ LP, Lipid peroxidation. ^bb^ HP, Hydrogen peroxide. ^cc^ PC, Protein carbonyls. ^dd^ SOD, Superoxide dismutase. ^ee^ GPx-4, Glutathione peroxidase 4. ^ff^ HR, Hydroxyl radicals. ^gg^ TBARS, Thiobarbituric acid reactive substances. ^hh^ CAT, Catalase. ^ii^ GST, Glutathione S-transferase. ^jj^ GR, Glutathione reductase. ^kk^ LOT, Lateral olfactory tract. ^ll^ HVA, Homovanillic acid. ^mm^ AOC, Acetone on chow. ^nn^ ST, Striatum. ^oo^ FC, Frontal cortex. ^pp^ 5-HT, Serotonin. ^qq^ 5-HIAA, 5-hydroxyindoleacetic acid. ^rr^ BS, Brainstem. ^ss^ HT, Hypothalamus. ^tt^ NE, Norepinephrine. ^uu^ DFC, Dorsal frontal cortex. ^vv^ MBH, Medial-basal hypothalamus. ^ww^ DR, Dopamine receptor. ^xx^ DRD1, Dopamine receptor D1. ^yy^ DRD5, Dopamine receptor D5. ^zz^ DRD2, Dopamine receptor D2. ^aaa^ DRD3, Dopamine receptor D3. ^bbb^ DRD4, Dopamine receptor D4. ^ccc^ TH, Tyrosine hydroxylase. ^ddd^ NF-kB, Nuclear factor kappa B. ^eee^ Bcl2, B-cell leukemia/lymphoma 2. ^fff^ Bid, BH3 interacting-domain death agonist. ^ggg^ Bad, BCL2-associated death promoter. ^hhh^ Bax, BCL2-associated X protein. ^iii^ CASP3, Caspase 3. ^jjj^ CASP9, Caspase 9. ^kkk^ TNFa, Tumor necrosis factor alpha. ^lll^ Fas, Tumor necrosis factor receptor superfamily member 6. ^mmm^ FasL, Fas ligand. ^nnn^ CASP8, Caspase 8. ^ooo^ AChE, Acetylcholinesterase. ^ppp^ GLAST, Glutamate-aspartate transporter. ^qqq^ BDNF, Brain-derived neurotrophic factor. ^rrr^ NTRKB, Neurotrophin tyrosine kinase receptor B. ^sss^ MAP1, Microtubule-associated protein. ^ttt^ ERK1, Extracellular signal-regulated kinase 1. ^uuu^ ERK2, Extracellular signal-regulated kinase 2. ^vvv^ CREB, Cyclic AMP response element binding protein. ^www^ GLT-1, Glutamate transporter 1. ^xxx^ SS, Synaptosomes. ^yyy^ GS, Gliosomes. ^zzz^ EAAC1, Excitatory amino acid carrier 1. ^aaaa^ ZO1, Zonula occludens-1. ^bbbb^ AF6, Afadin. ^cccc^ JAM, Junctional adhesion molecule. ^dddd^ Ras, Rat sarcoma. ^eeee^ Raf, Rapidly accelerated fibrosarcoma. ^ffff^ Nefl, Neurofilament light polypeptide.

**Table A3:** Summary of Studies Investigating Neurotoxic Outcomes in Long-Evans Rats Following PCB Exposure.

PCB Source	Dose (mg/kg/day) ^a^	Exposure Route	Vehicle	Exposure Period	Age	N ^b^	Sex	Brain Region	Analysis	Result ^c^	Ref
A1254 ^d^	0, 10 or 30	PO ^e^	CO ^f^	5 d/w ^g^, 4 weeks	PND60 ^h^	26, 25, 35	M ^i^	FC ^j^, CE ^k^, ST ^l^, MS ^m^, and MT ^n^	Ca^2+^ Buffering and PKC ^o^	↓ Ca^2+^ uptake in microsomes ↓ Ca^2+^ uptake in mitochondria of CE ↓ Total PKC activity in CE ↑ Membrane-bound PKC activity in CE	[114]
A1254	0, 10 or 30	PO	CO	5 d/w, 4 weeks	PND60	26, 25, 35	M	FC ST	Neurotransmitter Concentrations	- 5-HT ^p^, NE ^q^, DA ^r^, HVA ^s^ ↑ DOPAC ^t^ and 5-HIAA ^u^ in ST ↑ NE, DA, DOPAC, HVA, and 5-HIAA in ST vs. FC	[114]
A1254	0, 10 or 30	PO	CO	5 d/w, 4 weeks	PND60	26, 25, 35	M	ST	Tyrosine Hydroxylase	- TH ^v^ immunoreactivity - TH specific activity	[114]

^a^ Unless otherwise noted. ^b^ N, Number of animals per group and sex. ^c^ (-) no change; (↑) significant increase; (↓) significant decrease. ^d^ A, Aroclor. ^e^ PO, Oral. ^f^ CO, Corn Oil. ^g^ d/w, days per week. ^h^ PND, Postnatal day. ^i^ M, Male. ^j^ FC, Frontal cortex. ^k^ CE, Cerebellum. ^l^ ST, Striatum. ^m^ MS, Microsomes. ^n^ MT, Mitochondria. ^o^ PKC, Protein kinase C. ^p^ 5-HT, Serotonin. ^q^ NE, Norepinephrine. ^r^ DA, Dopamine. ^s^ HVA, Homovanillic acid. ^t^ DOPAC, 3,4-Dihydroxyphenylacetic acid. ^u^ 5-HIAA, 5-Hydroxyindoleacetic acid. ^v^ TH, Tyrosine Hydroxylase.

**Table A4:** Summary of Studies Investigating Neurotoxic Outcomes in C57Bl/6 Mice Following PCB Exposure.

PCB Source	Dose (mg/kg/day)	Exposure Route	Vehicle	Exposure Period	Age	N ^a^	Sex	Brain Region	Analysis	Result ^b^	Ref
A1254 ^c^	0, 6, 12 or 25	PO ^d^	SO ^e^ on VW ^f^	4 weeks	9 weeks	20	M ^f^	ST ^g^, CE ^h^	OS ^g^ Markers	↑ MDA ^h^, 4-HNE ^i^, PO ^j^ ↑ Accumulation in ST than CE	[120]
A1254	0, 6, 12 or 25	PO	SO on VW	4 weeks	9 weeks	20	M	ST, CE	OS Protein Expression	↑ HO-1 ^k^, MnSOD ^l^, CuZnSOD protein expression	[120]
A1254	0, 6, 12 or 25	PO	SO on VW	4 weeks	9 weeks	20	M	ST	DA ^m^ and Metabolites	↑ DA levels - DOPAC ^n^, HVA ^o^, HVA/DA ratio	[120]
A1254	0, 6, 12 or 25	PO	SO on VW	4 weeks	9 weeks	20	M	ST	TH ^p^ and DAT ^q^ Expression	↓ TH and DAT	[120]
A1254	0, 6, 12 or 25	PO	SO on VW	4 weeks	9 weeks	20	M	ST CE	Iron levels	↑ Iron and Iron+ cells in ST ↓ Iron levels CE - Iron staining CE	[120]
A1254	0, 6, 12 or 25	PO	SO on VW	4 weeks	9 weeks	20	M	ST	Iron Regulatory Protein Expression	↑ Ferritin levels at 7 days ↓ Ferritin levels at 14 days ↑ TfR1 ^r^ after 28 days	[120]
A1254	0, 6, 12 or 25	PO	SO on VW	4 weeks	9 weeks	20	M	SNPC ^s^ VTA ^t^	Neuronal cell number	↑ Cell death in TH+ and TH− cells	[120]

^a^ N, Number of animals per group and sex. ^b^ (-) no change; (↑) significant increase; (↓) significant decrease. ^c^ A, Aroclor. ^d^ PO, Oral. ^e^ SO, Soybean oil. ^f^ VW, Vanilla wafer. ^g^ OS, Oxidative stress. ^h^ MDA, Malondialdehyde. ^i^ 4-HNE, 4-Hydroxynonenal. ^j^ PO, Protein oxidation. ^k^ HO-1, Heme oxygenase. ^l^ SOD, Superoxide dismutase. ^m^ DA, Dopamine. ^n^ DOPAC, 3,4-Dihydroxyphenylacetic acid. ^o^ HVA, Homovanillic acid. ^p^ TH, Tyrosine hydroxylase. ^q^ DAT, Dopamine transporter. ^r^ TfR1, Transferrin receptor protein 1. ^s^ SNPC, Substantia nigra pars compacta. ^t^ VTA, Ventral tegmental area.

**Table A5:** Summary of Studies Investigating Neurobehavioral Outcomes in Wistar Rats Following PCB Exposure.

PCB Source	Dose (mg/kg/day) ^a^	Exposure Route	Vehicle	Exposure Period	Age	N ^b^	Sex	Behavioral Category	Analysis	Result ^c^	Ref
PCB 180	0, 19.4, 64.3, 194, 643, 2000, 6430 or 13,000 μg/kg/day	PO ^d^	CO ^e^	28 days	6 weeks	5	M ^f^ F ^g^	Locomotor and Exploration	Open Field Test	↑ Time and distance moved in inner zone in F ↓ Habituation across days 2–5 for time and distance moved in the inner zone ↑/↓ Quadratic relationship between dose and total distance moved - Habituation of total activity - Observed in M	[119]
A1221 ^h^	0 or 1	IP ^i^	4% DMSO ^j^	PND24 ^k^, 26, 28	PND24	VM ^l^: 12 EM ^m^: 10 VF ^n^: 10 EF ^o^: 12	M F	Anxiety	Juvenile Elevated Plus Maze	- Time spent on arms and number of entries into open/closed arms	[192]
A1221	0 or 1	IP	4% DMSO	PND24, 26, 28	PND24	VM: 12 EM: 10 VF: 10 EF: 12	M F	Anxiety	Adult Elevated Plus Maze	- Time spent on arms or number of entries into open/closed arms in adults exposed to juvenile PCB	[192]
A1254 A1221	45.5 ± 5.9 μg/m^3^	Inh ^p^	LA ^q^	6 d/w ^r^ for 13 weeks	Adult (241 ± 9 g)	S ^s^: 8 E ^t^: 12	F	Anxiety	Elevated Plus Maze	↑ Duration and number of entries in the closed arm	[25]
A1221	0 or 1	IP	4% DMSO	PND24, 26, 28	PND24	VM: 12 EM: 10 VF: 10 EF: 12	F M	Anxiety	Juvenile Light/Dark Box	- Time spent on the light side	[192]
A1221	0 or 1	IP	4% DMSO	PND24, 26, 28	PND24	VM: 12 EM: 10 VF: 10 EF: 12	F M	Anxiety	Adult Light/Dark Box	- Time spent on the light side	[192]
A1221	0 or 1	IP	4% DMSO	PND24, 26, 28	PND24	VM: 12 EM: 10 VF: 10 EF: 12	F M	Social and Sociosexual Behavior	Juvenile Sociability	- Time near stimulus - Choice of familiar or novel animal ↑ Latency to stimulus in F	[192]
A1221	0 or 1	IP	4% DMSO	PND24, 26, 28	PND24	VM: 12 EM: 10 VF: 10 EF: 12	F M	Social and Sociosexual Behavior	Adult Socio-sexual USV ^u^	- Number of USVs produced	[192]
A1221	0 or 1	IP	4% DMSO	PND24, 26, 28	PND24	VM: 12 EM: 10 VF: 10 EF: 12	F M	Social and Sociosexual Behavior	Adult Socio-sexual Choice	↑ Time in hormone stimulus zone ↑ Time with stimuli	[192]
A1248	0 or 1 mL oil containing 0.5 μg/g PCB	PO	COR ^v^	30 days	PND37	14	M	Operant Conditioning and Behavioral Flexibility	Operant Training Fixed Interval Presses	↑ Lever presses	[230]
A1248	0 or 1 mL oil containing 0.5 μg/g PCB	PO	COR	30 days	PND37	14	M	Operant Conditioning and Behavioral Flexibility	Operant Training Response Bursts	- Response bursts	[230]
A1248	0 or 1 mL oil containing 0.5 μg/g PCB	PO	COR	30 days	PND37	14	M	Operant Conditioning and Behavioral Flexibility	Operant Training Reinforcements Delivered	- Mean number of reinforcements delivered	[230]
A1248	0 or 1 mL oil containing 0.5 μg/g PCB	PO	COR	30 days	PND37	14	M	Operant Conditioning and Behavioral Flexibility	Operant Training Extinction Presses	- Mean number of lever presses	[230]
A1248	0 or 1 mL oil containing 0.5 μg/g PCB	PO	COR	30 days	PND37	14	M	Operant Conditioning and Behavioral Flexibility	Operant Training Transition Presses	↑ Lever presses in the first session than the second	[230]
A1248	0 or 562 ng/m^3^	Inh	LA	30 days	PND37	S: 23 E: 24	M F	Operant Conditioning and Behavioral Flexibility	Operant Training	↑ Bar pressing rates, differences over time, hyperactivity at 90 and 120 s, and number of response bursts ↑ Number of responses in males	[231]
A1254	62.1 ± 17.7 μg/m^3^	Inh	LA	28 days	Adult (239 ±8 g)	8	F	Learning and Memory	Morris Water Maze	- Escape latency ↓ Platform crossings	[24]
A1254 A1221	45.5 ± 5.9 μg/m^3^	Inh	LA	6 d/w for 13 weeks	Adult (241 ± 9 g)	S: 8 E: 12	F	Learning and Memory	Morris Water Maze	- Escape latency, travel distance, or time in target quadrant ↓ Platform crossings	[25]
A1221	0 or 1	IP	4% DMSO	PND24, 26, 28	PND24	VM: 12 EM: 10 VF: 10 EF: 12	F M	Social and Sociosexual Behavior	Affiliative USV	- Number of USVs produced	[192]
A1221	0 or 1	IP	4% DMSO	PND24, 26, 28	PND24	VM: 12 EM: 10 VF: 10 EF: 12	F M	Social and Sociosexual Behavior	Affiliative Behavior	↑ Latency to hop in F - Number of hops - Nape contacts	[192]

^a^ Unless otherwise noted. ^b^ N, Number of animals per group and sex. ^c^ (-) no change; (↑) significant increase; (↓) significant decrease. ^d^ PO, Oral. ^e^ CO, Corn oil. ^f^ M, Male. ^g^ F, Female. ^h^ A, Aroclor. ^i^ IP, Intraperitoneal injection. ^j^ DMSO, Dimethyl sulfoxide. ^k^ PND, Postnatal day. ^l^ VM, Vehicle male. ^m^ EM, Exposed male. ^n^ VF, Vehicle female. ^o^ EF, Exposed female. ^p^ Inh, Inhalation. ^q^ LA, Lab air. ^r^ d/w, Days per week. ^s^ S, Sham. ^t^ E, Exposed. ^u^ USV, Ultrasonic vocalization. ^v^ COR, Corn oil on chow.

**Table A6:** Summary of studies Investigating Neurobehavioral Outcomes in Sprague-Dawley Rats Following PCB Exposure.

PCB Source	Dose (mg/kg/day) ^a^	Exposure Route	Vehicle	Exposure Period	Age	N ^b^	Sex	Brain Region	Analysis	Result ^c^	Ref
A1254 ^d^	62.1 ± 17.7 μg/m^3^	Inh ^e^	LA ^f^	28 days	Adult (239 ± 8g)	8	F ^g^	Whole Brain	Histology H&E ^h^	- Gross histopathological changes in HC ^i^ - Features of necrosis or apoptosis	[24]
A1254 A1221	45.5 ± 5.9 μg/m^3^	Inh	LA	6 d/w ^j^ for 13 weeks	Adult (241 ± 9 g)	S ^k^: 8 E ^l^: 12	F	Whole Brain	Histology H&E	- Histopathological changes	[25]
A1254 ^m^	0, 10 or 30	PO ^n^	CO ^o^	5 d/w ^p^ for 4 weeks	PND60 ^q^	26, 25, 35	M ^r^	Locomotor and Exploration	Motor Activity	↓ Horizontal activity in 30 mg/kg/day -Vertical activity	[114]
A1254	0, 100, 300 or 1000	PO	CO	1 day	PND67	9	M	Locomotor and Exploration	Motor Activity	↓ Horizontal activity in 1000 mg/kg/day ↓ Vertical activity in 300 and 1000 mg/kg/day	[184]
A1254	0, 10, 30 or 100	PO	CO	5 d/w for 4 weeks ^s^	PND67	9	M	Locomotor and Exploration	Motor Activity	↓ Horizontal activity ↓ Vertical activity	[184]
A1254	0, 1, 3 or 10	PO	CO	5 d/w for 6 weeks	PND67	9	M	Locomotor and Exploration	Motor Activity	- Horizontal and vertical activity	[184]
A1221 A1254	0, 2.5 or 5	IP ^t^	SO ^u^	1 week	PND60	12	F ^v^	Reproductive and Maternal Behavior	Lordosis and Pacing Test	- Approach latency, mount return latency, intromission return latency, postejaculatory refractory period, and lordosis quotient	[238]
PCB 77	0 or 2	SQ ^w^	CO	GD6^x^-18	Adult	6	F	Reproductive and Maternal Behavior	Maternal Auto-grooming	- Time spent auto-grooming	[239]
PCB 77	0 or 2	SQ	CO	GD6-18	Adult	6	F	Reproductive and Maternal Behavior	Licking and Grooming Pups	↑ Time spent licking and grooming pups	[239]
PCB 77	0 or 2	SQ	CO	GD6-18	Adult	6	F	Reproductive and Maternal Behavior	Nursing Bouts	↑ Number of nursing bouts	[239]
PCB 77	0 or 2	SQ	CO	GD6-18	Adult	6	F	Reproductive and Maternal Behavior	Nursing	- Time nursing	[239]
PCB 77	0 or 2	SQ	CO	GD6-18	Adult	6	F	Reproductive and Maternal Behavior	Time on Nest	↑ Time spent on nest	[239]
PCB 77	0 or 2	SQ	CO	GD6-18	Adult	6	F	Reproductive and Maternal Behavior	High Crouch Nursing Posture	↓ Time in high crouch nursing posture	[239]
FRM ^y,z^	0, 3 or 6	PO	CO	PND27-50	PND27	14, 13, 14	F M	Cognitive Flexibility and Decision-Making	Set Shifting—Omissions and Response Latencies	↑ Average trial omissions in M - Position discrimination and position reversal omissions in M - Set shifting omissions	[248]
FRM	0, 3 or 6	PO	CO	PND27-50	PND27	14, 13, 14	F M	Cognitive Flexibility and Decision-Making	Set Shifting—Visual Cue Discrimination	↑ Errors to criterion in M - Errors to criterion in F	[248]
FRM	0, 3 or 6	PO	CO	PND27-50	PND27	14, 13, 14	F M	Cognitive Flexibility and Decision-Making	Set Shifting—Shift to Position	- Number of errors	[248]
FRM	0, 3 or 6	PO	CO	PND27-50	PND27	14, 13, 14	F M	Cognitive Flexibility and Decision-Making	Set Shifting—Response Reversal	↓ Number of errors in M - Number of errors in F	[248]
FRM	0, 3 or 6	PO	CO	PND27-50	PND27	14, 13, 14	F M	Cognitive Flexibility and Decision-Making	Set Shifting—Error Analyss	↓ Number of pervasive errors in M	[248]
FRM	0, 3 or 6	PO	CO	PND27-50	PND27	14, 13, 14	F M	Impulsivity and Timing	DRL ^aa^—Training	- Total presses or efficiency ratio	[248]
FRM	0, 3 or 6	PO	CO	PND27-50	PND27	14, 13, 14	F M	Impulsivity and Timing	DRL - DRL15 training	- Total presses or efficiency ratio	[248]
FRM	0, 3 or 6	PO	CO	PND27-50	PND27	14, 13, 14	F M	Impulsivity and Timing	DRL—Extinction	- Main effect of exposure or exposure × session interaction	[248]
A1254	0, 100 or 300	PO	CO	1 day	PND76	6	M	Learning and Memory	Flavor Aversion	↓ Saccharin intake - Fluid intake	[184]
A1254	0, 10 or 30	PO	CO	1 day	PND76	6	M	Learning and Memory	Flavor Aversion	↓ Saccharin intake - Fluid intake	[184]
A1254	0, 15 or 25	PO	CO	1 day	PND76	6	M	Learning and Memory	Flavor Aversion	↓ Saccharin intake ↓ Fluid intake	[184]
A1254	0, 7.5 or 15	PO	CO	14 days	PND76	6	M	Learning and Memory	Flavor Aversion	↓ Saccharin intake ↓ Fluid intake 15mg/kg	[184]
A1254	0, 3.5 or 7.5	PO	CO	14 days	PND76	6	M	Learning and Memory	Flavor Aversion	- Saccharin intake	[184]

^a^ Unless otherwise noted. ^b^ N, Number of animals per group and sex. ^c^ (-) no change; (↑) significant increase; (↓) significant decrease. ^d^ A, Aroclor. ^e^ Inh, Inhalation. ^f^ LA, Lab air. ^g^ F, Female. ^h^ H&E, Hematoxylin and eosin staining. ^i^ HC, Hippocampus. ^j^ d/w, Days per week. ^k^ S, Sham. ^l^ E, Exposed. ^m^ A, Aroclor. ^n^ PO, Oral. ^o^ CO, Corn oil. ^p^ d/w, Days per week. ^q^ PND, Postnatal day. ^r^ M, Male. ^s^ 100 mg/kg group only dosed for 2 weeks. ^t^ IP, Intraperitoneal injection. ^u^ SO, Soybean oil. ^v^ F, Female. ^w^ SQ, Subcutaneous injection. ^x^ GD, Gestational day. ^y^ FRM, Fox river mixture. ^z^ FRM mimics the congener profile found in walleye taken from the Fox River in northeast Wisconsin. FRM composition by mass %: 35% Aroclor 1242, 35% Aroclor 1248, 15% Aroclor 1254, 15% Aroclor 1260. ^aa^ DRL, Differential reinforcement of low rates of responding.

**Table A7:** Summary of Studies Investigating Neurobehavioral Outcomes in C57Bl/6 Mice and Swiss Albino Rats Following PCB Exposure.

PCB Source	Dose (mg/kg/day) ^a^	Exposure Route	Vehicle	Exposure Period	Age	N ^b^	Sex	Brain Region	Analysis	Result ^c^	Ref
C57Bl/6 Mice											
A1254 ^d^	0, 6, 12 or 25	PO ^e^	VWC ^f^ SO ^g^	4 weeks	9 weeks	20	M ^h^	Locomotor and Exploration	Locomotor Activity	↑ Vertical, horizontal, and ambulatory activity	[120]
Swiss Albino Mice											
Mixture ^i^ (PCB 28, 52, 101, 138, 153, 180)	0, 1, 10, or 100 ng/kg/day	PO	RO ^j^	GD ^k^ 0-21	11 weeks	10	F ^l^	Reproductive and Maternal Behavior	Next Building Activity	- Nest quality	[240]
Mixture (PCB 28, 52, 101, 138, 153, 180)	0, 1, 10, or 100 ng/kg/day	PO	RO	GD 0-21	11 weeks	10	F	Reproductive and Maternal Behavior	Pup Retrieving Test	- Latency period to retrieve pup and carry back to nest	[240]

^a^ Unless otherwise noted. ^b^ N, Number of animals per group and sex. ^c^ (-) no change; (↑) significant increase. ^d^ A, Aroclor. ^e^ PO, Oral. ^f^ VWC, Vanilla wafer cookie. ^g^ SO, Soybean oil. ^h^ M, Male. ^i^ Mixture composition by mass %: 2% PCB 28, 6% PCB 52, 12% PCB 101, 32% PCB 138, 37% PCB 153, 11% PCB 180. ^j^ RO, Rape oil. ^k^ GD, Gestational day. ^l^ F, Female.
