# Supplementary material for: A Scoping Review of Neurotoxic and Behavioral Outcomes Following Polychlorinated Biphenyl (PCB) Exposure in Post-Weaned Rodents
Source: Int J Mol Sci. 2025 Nov 7;26(22):10829. doi: 10.3390/ijms262210829 (PMC12652203; doi:10.3390/ijms262210829)
Supplement: Supplementary file 1 [file ijms-26-10829-s001.zip › ijms-3931778-supplementary.pdf]

SUPPLEMENTARY MATERIALS

A Scoping Review of Neurotoxic and Behavioral Outcomes Following Polychlorinated Biphenyl (PCB) Exposure in Rodents

Nicole M. Breese [1,2], Sophia G. Heim [1], Riley J. Samuelson [3] and Hans-Joachim Lehmler [1,2]

[1]Department of Occupational and Environmental Health, College of Public Health, The University of Iowa, Iowa City, Iowa 52242, USA, [2]Interdisciplinary Graduate Program in Human Toxicology, The University of Iowa, Iowa City, Iowa 52242, USA, [3]Hardin Library for the Health Sciences, University of Iowa Libraries, The University of Iowa, Iowa City, Iowa 52242, USA.

Corresponding Author: Dr. Hans-Joachim Lehmler
Department of Occupational and Environmental Health
The University of Iowa
University of Iowa Research Park, B164 MTF
Iowa City, IA 52242-5000
Phone: (319) 335-4310
Fax: (319) 335-4981

Table of Contents

Table S1. Search terms and categories used for the searches in PubMed, Embase, and Scopus. S5

Table S1. Search terms and categories used for the searches in PubMed, Embase, and Scopus.

Topics	PubMed	Embase	Scopus
Rodents	"Rats"[Mesh]<br>"Murinae"[Mesh]<br>"Mice"[Mesh]	'rat'/exp<br>'murine'/exp<br>'mouse'/exp<br>'Mus musculus'/exp<br>'Rattus rattus'/exp<br>'rodent'/exp<br>'pup (rodent)'/exp	Rodent<br>Rodents<br>Pup<br>Pups<br>“pup (rodent)”<br>Rat<br>Rats<br>Murinae<br>Rattus<br>Mus<br>“mus musculus”<br>Mouse<br>Mice<br>“rattus rattus”<br>murine
	Rodent[Title/Abstract]<br>Rodents[Title/Abstract]<br>Pup[Title/Abstract]<br>Pups[Title/Abstract]<br>“pup (rodent)”[Title/Abstract]<br>Rat[Title/Abstract]<br>Rats[Title/Abstract]<br>Murinae[Title/Abstract]<br>Rattus[Title/Abstract]<br>Mus[Title/Abstract]<br>“mus musculus”[Title/Abstract]<br>Mouse[Title/Abstract]<br>Mice[Title/Abstract]<br>“rattus rattus”[Title/Abstract]<br>murine[Title/Abstract]	Rodent:ti,ab,kw<br>rodents:ti,ab,kw<br>Pup:ti,ab,kw<br>Pups:ti,ab,kw<br>“pup (rodent)”:ti,ab,kw<br>Rat:ti,ab,kw<br>Rats:ti,ab,kw<br>Murinae:ti,ab,kw<br>Rattus:ti,ab,kw<br>Mus:ti,ab,kw<br>“mus musculus”:ti,ab,kw<br>Mouse:ti,ab,kw<br>Mice:ti,ab,kw<br>“rattus rattus”:ti,ab,kw<br>murine:ti,ab,kw	
Adolescent	"Adolescent"[Mesh]<br>"Child"[Mesh]<br>"Minors"[Mesh]	'adolescent'/exp<br>'juvenile'/exp<br>'child'/exp	Adolescen*<br>Teen*<br>Teen<br>Minor<br>Minors<br>Juvenile<br>Juveniles<br>Child*<br>child
	Adolescen*[Title/Abstract]<br>Teen*[Title/Abstract]<br>Teen[Title/Abstract]<br>Minor[Title/Abstract]<br>Minors[Title/Abstract]<br>Juvenile[Title/Abstract]<br>Juveniles[Title/Abstract]<br>Child*[Title/Abstract]<br>child[Title/Abstract]	Adolescen*:ti,ab,kw<br>Teen*:ti,ab,kw<br>Teen:ti,ab,kw<br>Minor:ti,ab,kw<br>Minors:ti,ab,kw<br>Juvenile:ti,ab,kw<br>Juveniles:ti,ab,kw<br>Child*:ti,ab,kw<br>child:ti,ab,kw	
Adult	“Adult”[Mesh]<br>“Middle Aged”[Mesh]<br>“Aging”[Mesh]<br>“Aged”[Mesh]<br>“Aged, 80 and over”[Mesh]<br>“Young Adult”[Mesh]	‘adult’/exp<br>‘middle aged’/exp<br>‘aging’/exp<br>‘aged’/exp<br>‘young adult’/exp<br>‘very elderly’/exp	Adult<br>Adults<br>“Middle Age”<br>“Middle Aged”<br>Aged<br>Ageing<br>Aging<br>Elder<br>Elders<br>Elderly<br>“Oldest Old”<br>“Young Adult”<br>“Young Adults”<br>Mature
	Adult[Title/Abstract]<br>Adults[Title/Abstract]<br>“Middle Age”[Title/Abstract]<br>“Middle Aged”[Title/Abstract]<br>Aged[Title/Abstract]<br>Ageing[Title/Abstract]<br>Aging[Title/Abstract]<br>Elder[Title/Abstract]<br>Elders[Title/Abstract]<br>Elderly[Title/Abstract]<br>“Oldest Old”[Title/Abstract]<br>“Young Adult”[Title/Abstract]<br>“Young Adults”[Title/Abstract]<br>Mature[Title/Abstract]	Adult:ti,ab,kw<br>Adults:ti,ab,kw<br>“Middle Age”:ti,ab,kw<br>“Middle Aged”:ti,ab,kw<br>Aged:ti,ab,kw<br>Ageing:ti,ab,kw<br>Aging:ti,ab,kw<br>Elder:ti,ab,kw<br>Elders:ti,ab,kw<br>Elderly:ti,ab,kw<br>“Oldest Old”:ti,ab,kw<br>“Young Adult”:ti,ab,kw<br>“Young Adults”:ti,ab,kw<br>Mature:ti,ab,kw	
PCB	"Polychlorinated Biphenyls"[Mesh]<br>"Biphenyl Compounds"[Mesh]<br>"Aroclors"[Mesh]	'polychlorinated biphenyl'/exp<br>'biphenyl derivative'/exp<br>'aroclor'/exp<br>'dioxin'/exp	PCB<br>PCBs<br>“Polychlorinated Biphenyls”<br>“Polychlorinated Biphenyl”

	"Dioxins and Dioxin-like Compounds"[Mesh]<br>"Dioxins"[Mesh]		“Biphenyl compound”<br>“biphenyl compounds”<br>Aroclor<br>Aroclors<br>"Dioxins and Dioxin-like Compounds"<br>Dioxin<br>Dioxins<br>“biphenyl derivative”
	PCB[Title/Abstract]<br>PCBs[Title/Abstract]<br>“Polychlorinated Biphenyls”[Title/Abstract]<br>“Polychlorinated Biphenyl”[Title/Abstract]<br>“Biphenyl compound”[Title/Abstract]<br>“biphenyl compounds’[Title/Abstract]<br>Aroclor[Title/Abstract]<br>Aroclors[Title/Abstract]<br>"Dioxins and Dioxin-like Compounds"[Title/Abstract]<br>Dioxin[Title/Abstract]<br>Dioxins[Title/Abstract]<br>“biphenyl derivative”[Title/Abstract]	PCB:ti,ab,kw<br>PCBs:ti,ab,kw<br>“Polychlorinated Biphenyls”:ti,ab,kw<br>“Polychlorinated Biphenyl”:ti,ab,kw<br>“Biphenyl compound”:ti,ab,kw<br>“biphenyl compounds”:ti,ab,kw<br>Aroclor:ti,ab,kw<br>Aroclors:ti,ab,kw<br>"Dioxins and Dioxin-like Compounds":ti,ab,kw<br>Dioxin:ti,ab,kw<br>Dioxins:ti,ab,kw<br>“biphenyl derivative”:ti,ab,kw	
Neuro	"Attention Deficit Disorder with Hyperactivity"[Mesh]<br>"Neurodevelopmental Disorders"[Mesh]<br>"toxicity"[Subheading]<br>"Neurocognitive Disorders"[Mesh]<br>"Growth and Development"[Mesh]<br>"Brain"[Mesh]<br>"Central Nervous System"[Mesh]<br>"Nervous System"[Mesh]	'attention deficit disorder'/exp<br>'mental disease'/exp<br>'toxicity'/exp<br>'growth, development and aging'/exp<br>'brain'/exp<br>'central nervous system'/exp<br>'nervous system'/exp	"Attention Deficit Disorder with Hyperactivity"<br>“Attention Deficit Disorder”<br>ADHD<br>ADD<br>Toxic<br>Toxi*<br>“neurodevelopment disorders”<br>“neurodevelopment disorder”<br>Neurotox*<br>Neurodeve*<br>"Neurocognitive Disorders"<br>"Neurocognitive Disorder"<br>“Mental disease”<br>Neurocogn*<br>"Growth and Development"<br>Growth<br>Develop*<br>Develop<br>Brain<br>Brains<br>"Central Nervous System"<br>CNS<br>"Nervous System"<br>“growth, development and aging”<br>“attention deficit hyperactivity disorder”
	"Attention Deficit Disorder with Hyperactivity"[Title/Abstract]<br>“Attention Deficit Disorder”[Title/Abstract]<br>ADHD[Title/Abstract]<br>ADD[Title/Abstract]<br>Toxic[Title/Abstract]<br>Toxi*[Title/Abstract]<br>“neurodevelopment disorders”[Title/Abstract]<br>“neurodevelopment disorder”[Title/Abstract]<br>Neurotox*[Title/Abstract]<br>Neurodeve*[Title/Abstract]<br>"Neurocognitive Disorders"[Title/Abstract]<br>"Neurocognitive Disorder"[Title/Abstract]<br>“Mental disease”[Title/Abstract]<br>Neurocogn*[Title/Abstract]<br>"Growth and Development"[Title/Abstract]<br>Growth[Title/Abstract]<br>Develop*[Title/Abstract]<br>Develop[Title/Abstract]<br>Brain[Title/Abstract]<br>Brains[Title/Abstract]<br>"Central Nervous System"[Title/Abstract]<br>CNS[Title/Abstract]<br>"Nervous System"[Title/Abstract]<br>“growth, development and aging”[Title/Abstract]<br>“attention deficit hyperactivity disorder”[Title/Abstract]	"Attention Deficit Disorder with Hyperactivity":ti,ab,kw<br>“Attention Deficit Disorder”:ti,ab,kw<br>ADHD:ti,ab,kw<br>ADD:ti,ab,kw<br>Toxic:ti,ab,kw<br>Toxi*:ti,ab,kw<br>“neurodevelopment disorders”:ti,ab,kw<br>“neurodevelopment disorder”:ti,ab,kw<br>Neurotox*:ti,ab,kw<br>Neurodeve*:ti,ab,kw<br>"Neurocognitive Disorders":ti,ab,kw<br>"Neurocognitive Disorder":ti,ab,kw<br>“Mental disease”:ti,ab,kw<br>Neurocogn*:ti,ab,kw<br>"Growth and Development":ti,ab,kw<br>Growth:ti,ab,kw<br>Develop*:ti,ab,kw<br>Develop:ti,ab,kw<br>Brain:ti,ab,kw<br>Brains:ti,ab,kw<br>"Central Nervous System":ti,ab,kw<br>CNS:ti,ab,kw<br>"Nervous System":ti,ab,kw<br>“growth, development and aging”:ti,ab,kw<br>“attention deficit hyperactivity disorder”:ti,ab,kw	

Initial PubMed Search strategy
N=1796 (11/20/2023)

("Rats"[Mesh] OR "Murinae"[Mesh] OR "Mice"[Mesh] OR Rodent[Title/Abstract] OR Rodents[Title/Abstract] OR Pup[Title/Abstract] OR Pups[Title/Abstract] OR “pup (rodent)”[Title/Abstract] OR Rat[Title/Abstract] OR Rats[Title/Abstract] OR Murinae[Title/Abstract] OR Rattus[Title/Abstract] OR Mus[Title/Abstract] OR “mus musculus”[Title/Abstract] OR Mouse[Title/Abstract] OR Mice[Title/Abstract] OR “rattus rattus”[Title/Abstract] OR murine[Title/Abstract]) AND ("Adolescent"[Mesh] OR "Child"[Mesh] OR "Minors"[Mesh] OR Adolescen*[Title/Abstract] OR Teen*[Title/Abstract] OR Teen[Title/Abstract] OR Minor[Title/Abstract] OR Minors[Title/Abstract] OR Juvenile[Title/Abstract] OR Juveniles[Title/Abstract] OR Child*[Title/Abstract] OR child[Title/Abstract] OR “Adult”[Mesh] OR “Middle Aged”[Mesh] OR “Aging”[Mesh] OR “Aged”[Mesh] OR “Aged, 80 and over”[Mesh] OR “Young Adult”[Mesh] OR Adult[Title/Abstract] OR Adults[Title/Abstract] OR “Middle Age”[Title/Abstract] OR “Middle Aged”[Title/Abstract] OR Aged[Title/Abstract] OR Ageing[Title/Abstract] OR Aging[Title/Abstract] OR Elder[Title/Abstract] OR Elders[Title/Abstract] OR Elderly[Title/Abstract] OR “Oldest Old”[Title/Abstract] OR “Young Adult”[Title/Abstract] OR “Young Adults”[Title/Abstract] OR Mature[Title/Abstract]) AND ("Polychlorinated Biphenyls"[Mesh] OR "Biphenyl Compounds"[Mesh] OR "Aroclors"[Mesh] OR "Dioxins and Dioxin-like Compounds"[Mesh] OR "Dioxins"[Mesh] OR PCB[Title/Abstract] OR PCBs[Title/Abstract] OR “Polychlorinated Biphenyls”[Title/Abstract] OR “Polychlorinated Biphenyl”[Title/Abstract] OR “Biphenyl compound”[Title/Abstract] OR “biphenyl compounds”[Title/Abstract] OR Aroclor[Title/Abstract] OR Aroclors[Title/Abstract] OR "Dioxins and Dioxin-like Compounds"[Title/Abstract] OR Dioxin[Title/Abstract] OR Dioxins[Title/Abstract] OR “biphenyl derivative”[Title/Abstract]) AND ("Attention Deficit Disorder with Hyperactivity"[Mesh] OR "Neurodevelopmental Disorders"[Mesh] OR "toxicity"[Subheading] OR "Neurocognitive Disorders"[Mesh] OR "Growth and Development"[Mesh] OR "Brain"[Mesh] OR "Central Nervous System"[Mesh] OR "Nervous System"[Mesh] OR "Attention Deficit Disorder with Hyperactivity"[Title/Abstract] OR “attention deficit hyperactivity disorder”[Title/Abstract] OR “Attention Deficit Disorder”[Title/Abstract] OR ADHD[Title/Abstract] OR ADD[Title/Abstract] OR Toxic[Title/Abstract] OR Toxi*[Title/Abstract] OR “neurodevelopment disorders”[Title/Abstract] OR “neurodevelopment disorder”[Title/Abstract] OR Neurotox*[Title/Abstract] OR Neurodeve*[Title/Abstract] OR "Neurocognitive Disorders"[Title/Abstract] OR "Neurocognitive Disorder"[Title/Abstract] OR “Mental disease”[Title/Abstract] OR Neurocogn*[Title/Abstract] OR "Growth and Development"[Title/Abstract] OR Growth[Title/Abstract] OR Develop*[Title/Abstract] OR Develop[Title/Abstract] OR Brain[Title/Abstract] OR Brains[Title/Abstract] OR "Central Nervous System"[Title/Abstract] OR CNS[Title/Abstract] OR "Nervous System"[Title/Abstract] OR “growth, development and aging”[Title/Abstract])

Initial EMBASE Search strategy
N=2787 (11/20/2023)
('rat'/exp OR 'murine'/exp OR 'mouse'/exp OR 'Mus musculus'/exp OR 'Rattus rattus'/exp OR 'rodent'/exp OR 'pup (rodent)'/exp OR Rodent:ti,ab,kw OR rodents:ti,ab,kw OR Pup:ti,ab,kw OR Pups:ti,ab,kw OR “pup (rodent)”:ti,ab,kw OR Rat:ti,ab,kw OR Rats:ti,ab,kw OR Murinae:ti,ab,kw OR Rattus:ti,ab,kw OR Mus:ti,ab,kw OR “mus musculus”:ti,ab,kw OR Mouse:ti,ab,kw OR Mice:ti,ab,kw OR “rattus rattus”:ti,ab,kw OR murine:ti,ab,kw) AND ('adolescent'/exp OR 'juvenile'/exp OR 'child'/exp OR Adolescen*:ti,ab,kw OR Teen*:ti,ab,kw OR Teen:ti,ab,kw OR Minor:ti,ab,kw OR Minors:ti,ab,kw OR Juvenile:ti,ab,kw OR Juveniles:ti,ab,kw OR Child*:ti,ab,kw OR child:ti,ab,kw OR ‘adult’/exp OR ‘middle aged’/exp OR ‘aging’/exp OR ‘aged’/exp OR ‘young adult’/exp OR ‘very elderly’/exp OR Adult:ti,ab,kw OR Adults:ti,ab,kw OR “Middle Age”:ti,ab,kw OR “Middle Aged”:ti,ab,kw OR Aged:ti,ab,kw OR Ageing:ti,ab,kw OR Aging:ti,ab,kw OR Elder:ti,ab,kw OR Elders:ti,ab,kw OR Elderly:ti,ab,kw OR “Oldest Old”:ti,ab,kw OR “Young Adult”:ti,ab,kw OR “Young Adults”:ti,ab,kw OR Mature:ti,ab,kw) AND ('polychlorinated biphenyl'/exp OR 'biphenyl derivative'/exp OR 'aroclor'/exp OR 'dioxin'/exp OR PCB:ti,ab,kw OR PCBs:ti,ab,kw OR “Polychlorinated Biphenyls”:ti,ab,kw OR “Polychlorinated Biphenyl”:ti,ab,kw OR “Biphenyl compound”:ti,ab,kw OR “biphenyl compounds”:ti,ab,kw OR Aroclor:ti,ab,kw OR Aroclors:ti,ab,kw OR "Dioxins and Dioxin-like Compounds":ti,ab,kw OR Dioxin:ti,ab,kw OR Dioxins:ti,ab,kw OR “biphenyl derivative”:ti,ab,kw) AND ('attention deficit disorder'/exp OR 'mental disease'/exp OR 'toxicity'/exp OR 'growth, development and aging'/exp OR 'brain'/exp OR 'central nervous system'/exp OR 'nervous system'/exp OR “Attention Deficit Disorder with Hyperactivity":ti,ab,kw OR “attention deficit hyperactivity disorder”:ti,ab,kw OR “Attention Deficit Disorder”:ti,ab,kw OR ADHD:ti,ab,kw OR ADD:ti,ab,kw OR Toxic:ti,ab,kw OR Toxi*:ti,ab,kw OR “neurodevelopment disorders”:ti,ab,kw OR “neurodevelopment disorder”:ti,ab,kw OR Neurotox*:ti,ab,kw OR Neurodeve*:ti,ab,kw OR "Neurocognitive Disorders":ti,ab,kw OR "Neurocognitive Disorder":ti,ab,kw OR “Mental disease”:ti,ab,kw OR Neurocogn*:ti,ab,kw OR "Growth and Development":ti,ab,kw OR Growth:ti,ab,kw OR Develop*:ti,ab,kw OR Develop:ti,ab,kw OR Brain:ti,ab,kw OR Brains:ti,ab,kw OR "Central Nervous System":ti,ab,kw OR CNS:ti,ab,kw OR "Nervous System":ti,ab,kw OR “growth, development and aging”:ti,ab,kw)

Initial SCOPUS Search strategy
N=1583 (11/20/2023)
((TITLE-ABS-KEY (rodent) OR TITLE-ABS-KEY (rodents) OR TITLE-ABS-KEY (pup) OR TITLE-ABS-KEY (pups) OR TITLE-ABS-KEY (rat) OR TITLE-ABS-KEY (rats) OR TITLE-ABS-KEY (murinae) OR TITLE-ABS-KEY (rattus) OR TITLE-ABS-KEY (mus) OR TITLE-ABS-KEY ("mus musculus") OR TITLE-ABS-KEY (mouse) OR TITLE-ABS-KEY (mice) OR TITLE-ABS-KEY ("rattus rattus") OR TITLE-ABS-KEY (murine))) AND ((TITLE-ABS-KEY (adolescen*) OR TITLE-ABS-KEY (teen*) OR TITLE-ABS-KEY (teen) OR TITLE-ABS-KEY (minor) OR TITLE-ABS-KEY (minors) OR TITLE-ABS-KEY (juvenile) OR TITLE-ABS-KEY (juveniles) OR TITLE-ABS-KEY (child*) OR TITLE-ABS-KEY (child) OR TITLE-ABS-KEY (Adult) OR TITLE-ABS-KEY (Adults) OR TITLE-ABS-KEY (“Middle Age”) OR TITLE-ABS-KEY (“Middle Aged”) OR TITLE-ABS-KEY (Aged) OR TITLE-ABS-KEY (Ageing) OR TITLE-ABS-KEY (Aging) OR TITLE-ABS-KEY (Elder) OR TITLE-ABS-KEY (Elders) OR TITLE-ABS-KEY (Elderly) OR TITLE-ABS-KEY (“Oldest Old”) OR TITLE-ABS-KEY (“Young Adult”) OR TITLE-ABS-KEY (“Young Adults”) OR TITLE-ABS-KEY (Mature))) AND ((TITLE-ABS-KEY (pcb) OR TITLE-ABS-KEY (pcbs) OR TITLE-ABS-KEY ("polychlorinated biphenyls") OR TITLE-ABS-KEY ("polychlorinated biphenyl") OR TITLE-ABS-KEY ("biphenyl compound") OR TITLE-ABS-KEY ("biphenyl compounds") OR TITLE-ABS-KEY (aroclor) OR TITLE-ABS-KEY (aroclors) OR TITLE-ABS-KEY ("Dioxins and Dioxin-like Compounds") OR TITLE-ABS-KEY (dioxin) OR TITLE-ABS-KEY (dioxins) OR TITLE-ABS-KEY ("biphenyl derivative"))) AND ((TITLE-ABS-KEY ("Attention Deficit Disorder with Hyperactivity") OR TITLE-ABS-KEY ("Attention Deficit Disorder") OR TITLE-ABS-KEY (“attention deficit hyperactivity disorder”) OR TITLE-ABS-KEY (adhd) OR TITLE-ABS-KEY (add) OR TITLE-ABS-KEY (toxic) OR TITLE-ABS-KEY (toxi*) OR TITLE-ABS-KEY ("neurodevelopment disorders") OR TITLE-ABS-KEY ("neurodevelopment disorder") OR TITLE-ABS-KEY (neurotox*) OR TITLE-ABS-KEY (neurodeve*) OR TITLE-ABS-KEY ("neurocognitive disorders") OR TITLE-ABS-KEY ("neurocognitive disorder") OR TITLE-ABS-KEY ("mental disease") OR TITLE-ABS-KEY (neurocogn*) OR TITLE-ABS-KEY ("growth and development") OR TITLE-ABS-KEY (growth) OR TITLE-ABS-KEY (develop*) OR TITLE-ABS-KEY (brain) OR TITLE-ABS-KEY (brains) OR TITLE-ABS-KEY (develop) OR TITLE-ABS-KEY ("central nervous system") OR TITLE-ABS-KEY (cns) OR TITLE-ABS-KEY ("nervous system") OR TITLE-ABS-KEY ("growth, development and aging"))) AND (LIMIT-TO (LANGUAGE , "English"))
